# Human BST2 inhibits rabies virus release independently of cysteine-linked dimerization and asparagine-linked glycosylation

**DOI:** 10.1371/journal.pone.0292833

**Published:** 2023-11-03

**Authors:** Nathiphat Tanwattana, Nanchaya Wanasen, Yuparat Jantraphakorn, Kanjana Srisutthisamphan, Thanathom Chailungkarn, Suwimon Boonrungsiman, Boonlert Lumlertdacha, Porntippa Lekchareonsuk, Challika Kaewborisuth

**Affiliations:** 1 Interdisciplinary Program in Genetic Engineering and Bioinformatics, Graduate School, Kasetsart University, Bangkok, Thailand; 2 Virology and Cell Technology Research Team, National Center for Genetic Engineering and Biotechnology (BIOTEC), National Science and Technology Development Agency (NSTDA), Pathumthani, Thailand; 3 National Nanotechnology Center (NANOTEC), National Science and Technology Development Agency (NSTDA), KlongLuang, Pathum Thani, Thailand; 4 Queen Saovabha Memorial Institute, Thai Red Cross Society, WHO Collaborating Center for Research and Training Prophylaxis on Rabies, Pathumwan, Bangkok, Thailand; 5 Department of Microbiology and Immunology, Faculty of Veterinary Medicine, Kasetsart University, Bangkok, Thailand; 6 Center for Advance Studies in Agriculture and Food, KU Institute Studies, Kasetsart University, Bangkok, Thailand; Nagasaki University, JAPAN

## Abstract

The innate immune response is a first-line defense mechanism triggered by rabies virus (RABV). Interferon (IFN) signaling and ISG products have been shown to confer resistance to RABV at various stages of the virus’s life cycle. Human tetherin, also known as bone marrow stromal cell antigen 2 (hBST2), is a multifunctional transmembrane glycoprotein induced by IFN that has been shown to effectively counteract many viruses through diverse mechanisms. Here, we demonstrate that hBST2 inhibits RABV budding by tethering new virions to the cell surface. It was observed that release of virus-like particles (VLPs) formed by RABV G (RABV-G VLPs), but not RABV M (RABV-G VLPs), were suppressed by hBST2, indicating that RABV-G has a specific effect on the hBST2-mediated restriction of RABV. The ability of hBST2 to prevent the release of RABV-G VLPs and impede RABV growth kinetics is retained even when hBST2 has mutations at dimerization and/or glycosylation sites, making hBST2 an antagonist to RABV, with multiple mechanisms possibly contributing to the hBST2-mediated suppression of RABV. Our findings expand the knowledge of host antiviral mechanisms that control RABV infection.

## Introduction

Rabies virus (RABV) is a member of the *Lyssavirus* genus, which is part of the *Rhabdoviridae* family. It causes rabies, a severe neurological disease with a 100% fatality rate that kills approximately 60,000 people worldwide each year [1], despite the availability of prophylactic vaccines. Post-infection use of therapeutic vaccines only provides protection against the disease before symptoms appear. RABV has a negative-sense RNA genome of approximately 12 kb in length. Its genome encodes five viral proteins, namely nucleoprotein (N), phosphoprotein (P), matrix protein (M), glycoprotein (G), and a large polymerase protein (L). RABV N, P and L form a ribonucleoprotein core which encapsulates the viral RNA genome (RNP) and play crucial roles in regulating viral genome transcription and replication [2, 3]. RABV P associates with host proteins to promote efficient viral production, spread through neurons [3, 4], and viral pathogenesis [3, 5]. RABV M helps bridge RNPs and the cytoplasmic domain of RABV G to support virion structure [6]. RABV G is a key virulence factor responsible for innate immune induction, viral neurotropism and neuroinvasiveness [7].

In terms of virus-host interactions, RABV infection activates host innate antiviral pathways. After viral entry and genome transcription, host cells recognize viral RNA intermediates via pattern-recognition receptors (PRRs) such as Toll-like receptors (TLRs), RIG-I-like receptors (RLRs), and NOD-like receptors (NLRs). This triggers the activation of downstream signaling molecules, primarily type I IFNs, which turn on potent IFN-inducible genes (ISGs) that are crucial in defending cells against viral invasion. Some of these ISGs, including viperin [8], interferon-inducible GTPase 1 (IIGP1) [9], and interferon-induced protein with tetratricopeptide repeats 2 (IFIT2) [10] have been revealed to limit RABV replication *in vitro* and *in vivo*. Investigating the interplay between host restriction factors and RABV is important for developing post-exposure treatments.

BST2, also known as bone marrow stromal antigen 2, CD317, HM1.24, or tetherin, is a well-known antiviral ISG. This type II transmembrane protein comprises an N-terminal cytoplasmic tail (NT), a single transmembrane region (TM), a coiled-coil ectodomain (ED), and a glycosylphosphatidylinositol (GPI) anchor at the C-terminus. The NT region contains a YxY motif, which regulates the innate immune pathway and is involved in NF-kB activation [11, 12]. The TM and GPI domains associate with the ER membrane and trans-Golgi network (TGN) and plasma membrane, respectively [13, 14]. The ED region has three critical cysteine-dimerization and two N-linked glycosylation sites essential for BST2’s antiviral functions against the HIV-1 virus [15, 16]. BST2 localization in the plasma membrane, trans-Golgi, and early/recycling endosomes [17] may enable its interaction with viral surface proteins, thus limiting their transport. BST2 has been shown to tether virions to the cell surface. This mechanism is shared by many viruses, including HIV-1 [17], Lassa virus [18, 19], herpesviruses [20], filovirus-based VLPs [21, 22], influenza A virus [23], and vesicular stomatitis virus (VSV) [24].

While BST2 can be induced to suppress viral replication, many viral proteins can interfere with BST2’s functions. For example, the HIV-1 Vpu protein binds to BST2 and transports it to lysosomes for degradation [25]. The Ebola virus glycoprotein (GP) has also been found to counter hBST2 restriction via a direct interaction with hBST2, consequently, restoring efficient virion budding [22]. Several studies have also shown that BST2 antagonists promote viral pathogenesis and disease progression after virus challenge *in vivo* [26]. BST2-mediated resistance to rhabdovirus infection has been observed in several aspects, especially against VSV, where BST2 has been shown to reduce VSV titer by three logs in HEK293T cell culture [24]. The discovery implies that BST2 plays a crucial role in regulating rhabdovirus infection; however, the mechanisms and evidence by which BST2 can inhibit RABV replication are still unclear. To enhance our understanding on BST2-rhabdovirus interplay, we investigated the effects of human BST2 (hBST2) on RABV release and production.

## Materials and methods

### Cells and virus

BHK21.C13 (*Mesocricetus auratus*-derived kidney cells; ATCC^®^ CCL-10) and HEK293T (ATCC^®^ CRL-3216) cells were maintained in Opti-MEM^TM^ media (Thermofisher Scientific) supplemented with 10% fetal bovine serum (FBS) (Thermofisher Scientific) and incubated at 37°C in a humidified atmosphere at 5% CO_2_.

Queen Saovabha Memorial Institute (Bangkok, Thailand) provided a Thai RABV strain (RABV). The virus was isolated from the brain of a rabid dog, propagated in mouse brains for seven passages, and then cultured in BHK21.C13 cells for five passages to increase virus titers.

### Plasmid construction

Individual RABV protein including G, M, N and P with a myc-tag at the C-terminus and wild-type hBST2 (hBST2) with a flag-tag at the N-terminus were codon-optimized and cloned into pCAGGS plasmid. The hBST2 mutants including mutations in dimerization (C3A) or glycosylation (N2A) site and double mutations (C3AN2A) were generated by site-directed mutagenesis using Q5^®^ Site-Directed Mutagenesis Kit (NEB) following the manufacturer’s instructions. Each hBST2 gene (wild-type and mutants) was inserted into the pSIN-CSGW-UbEm under the spleen focus-forming virus (SFFV) promoters (pUbEm-hBST2) and used for a co-transfection with lentivirus plasmids to rescue hBST2-expressing lentivirus used for a generation of a cell line stably expressing hBST2.

### Generation of hBST2 expressing cell lines

To generate BHK21.C13 cells stably expressing hBST2 (BHK21.hBST2, BHK21.hBST2_C3A_, BHK21.hBST2_N2A_ and BHK21.hBST2_C3AN2A_), the cells were transduced with lentivirus carrying each hBST2 gene. Briefly, HEK293T cells were seeded in a 6-well plate and transfected with 1.5 μg pUbEm-hBST2, 1.0 μg pCMVΔ8.91, and 0.5 μg pMD.G complexed with Fugene^®^ HD (Promega) at 1:3 ratio (DNA:Fugene^®^ HD). Three days after transfection, the supernatant containing recombinant lentivirus harboring the hBST2 gene (lenti-Em-hBST2) was harvested and used for transduction of BHK21.C13 cells in the presence of 10 μg/mL polybrene. Seventy-two hours post-transduction, transduced cells were sorted for green-fluorescent cells on a FACSAria^TM^ Fusion flow cytometer (BD Biosciences). hBST2 positive clones (BHK21.hBST2 and mutants) were expanded and used for RABV infection. Lentivirus reporter control was used to transduce BHK21.C13 cells to generate BHK21.C13 hBST2 negative cells (BHK21 control).

Cell viability as measured by cell counting (trypan blue exclusion test) and WST-1 assay (Promega) was examined to ensure that all cell types (WT-BHK21, BHK21.control and hBST2-expressing BHK21 cells) were healthy and proliferated at similar levels before being used for RABV infection.

### WST-1 metabolic activity assay

Wild-type BHK21 (WT-BHK21), BHK21.control and hBST2-expressing BHK21 cells were prepared at a density of 1×10^5^ cells in 6-well plates. Metabolic activity in BHK21 cells was measured at various time points (24–72 hours) after cell seeding. Ten microliters of cell proliferation reagent WST-1 (Promega) were added into each well. Plates were incubated for 2 hours at 37°C, 5% CO_2_. Absorbance was measured at 450 nm in a Synergy HTX microplate reader (BioTek). Each sample was prepared in triplicate.

### RABV infection

BHK21 or hBST2-expressing BHK21 cells were seeded in 6-well plates (2.0 x 10^5^ cells/well) and infected in triplicate with RABV at an MOI of 0.01. After 1 hour of virus adsorption, cells were washed three times with phosphate-buffered saline (PBS) and maintained in Opti-MEM^TM^ media (Thermofisher Scientific) supplemented with 2% FBS (Thermofisher Scientific) at 37°C in a humidified atmosphere at 5% CO_2_. At 0, 24, 48, 72, and 96 hours post-infection (hpi), supernatants containing RABV particles were collected and subjected to virus titration and RT-qPCR to quantify RABV infectious particles and viral RNA, respectively.

BHK21 and hBST2-expressing BHK21 cells were infected with RABV at an MOI of 1 in triplicate to examine intra- and extracellular RABV titers. Cell supernatants and pellets were collected after 8, 24, and 48 hours. Virus particles in the cell pellet fraction (in 200 μl Opti-MEM^TM^) were obtained after three freeze-thaw cycles (liquid nitrogen-room temperature cycles) and supernatants were collected by centrifugation at 5,000 x g for 10 minutes. To quantify RABV infectious particles and viral RNA, the supernatants were subjected to the 50% tissue culture infectious dose (TCID50) assay and RT-qPCR, respectively.

### Virus titration

TCID50 assay was determined as previously described [27]. Ten-fold dilutions of the infectious supernatants were prepared in quadruplicate in Opti-MEM^TM^ media. Fifty microliters of diluted samples were transferred into each well of a 96-well plate seeded with BHK21.C13 cells. After an hour of incubation at 37°C, the media was removed and replaced with 100 μl of fresh Opti-MEM^TM^ media. The cells were then incubated for 72 hours before being fixed for 5 minutes in cold 80% acetone. Fixed cells were washed twice with PBS before being incubated for 1 hour at room temperature with horse α-RABV serum (Queen Saovabha Memorial Institute). After washing three times with PBS plus 0.05% Tween20 (PBST), the cells were incubated for 1 hour at room temperature with goat α-horse IgG Dylight-488 (Abcam) antibodies before being washed with PBST. The cells were observed under a fluorescence microscope (Olympus). Virus titers were calculated using the Reed and Muench method and expressed as TCID50/ml [28].

### RT-qPCR

Viral RNAs (vRNAs) were isolated from cell supernatants using the Viral Nucleic Acid Extraction Kit II (Geneaid, Taiwan) following the manufacturer’s protocol. vRNAs or cellular total RNAs were extracted from cell pellets using the GeneJET RNA Purification Kit (Thermofisher Scientific) following the manufacturer’s protocol. To quantify RABV vRNA or BST2 mRNA in RABV-infected BHK21 cells, 500 ng of total RNAs were reverse transcribed with a specific primer to vRNA (GATAGAGCAGATTTTTGA) or oligo-dT (Thermofisher Scientific), respectively, using the RevertAid RT Reverse Transcription Kit (Thermofisher Scientific).

Quantitative real-time PCR (qPCR) was performed using the CFX96 Touch Real-Time PCR Detection System (Bio-Rad). Briefly, forward and reverse primers specific for RABV N or BST2 and cDNA were mixed with iTaq™ Universal SYBR^®^ Green Supermix (Bio-Rad) following the manufacturer’s instructions. The qPCR was conducted under the following conditions: initial denaturation at 95°C for 30 seconds followed by 40 cycles of denaturation at 95°C for 5 seconds and annealing and extension at 60°C for 30 seconds. Absolute vRNAs (copies/ml) were calculated from a standard curve of 10-fold serial dilutions of the RABV cDNA standard. Housekeeping β-actin gene was used as an internal control for BST2 mRNA normalization.

### Peptide-N-glycosidase-F (PNGase F) treatment

HEK293T cells were seeded in 6-well plate (6×10^5^ cells/well) and transfected with 1 μg of plasmids expressing flaghBST2 using Fugene® HD (Promega). At 24 hours post-transfection (hpt), cleared cell lysates were collected by centrifuging at 10,000 rpm for 5 mins and lysed with 100 μl of mammalian lysis buffer (Thermofisher Scientific) supplemented with protease inhibitor cocktail (Thermofisher Scientific). The samples were treated with PNGase F (NEB) following the manufacturers’ instruction with slight modifications. Briefly, 25 μl of sample was mixed with 1X denaturing buffer in a 30 μl total reaction volume. The mixture was boiled for 10 mins and chilled on ice for 1 min. Four microliters of Glycobuffer 2, 4 μl of 10% NP-40, 1 μl of PNGase F and 1 μl of distilled water were then added making a 40 μl total reaction volume. The reaction was incubated at 37°C overnight. The extent of deglycosylation of hBST2 in treated samples was examined by mobility shifts on SDS-PAGE gel. Western blot analysis was performed to visualize hBST2 on nitrocellulose membrane using rabbit α-flag and goat α-rabbit IgG HRP antibodies.

### Scanning electron microscopy (SEM)

BHK21 and BHK21.hBST2 cells were infected with RABV at an MOI of 1 for 24 hours. The cells were fixed with 4% (v/v) glutaraldehyde in 0.1 M PIPES buffer at 4°C for 1 hour and washed. The cells were then fixed with 1% (w/v) osmium tetroxide made in 0.1 M PIPES buffer for 1 hour at room temperature and washed thoroughly. Specimens were dehydrated in graded concentrations of ethanol from 50%, 70%, 90% to 100%, dried using critical point dryer (EM CPD300, Leica), and gold coated (EM ACE600 coater, Leica) before SEM observation. SEM study was performed on the Field Emission Scanning Electron microscope (Versa 3D, FEI), operated at 10 kV.

### Confocal microscopy

HEK293T cells transfected with plasmids expressing individual RABV proteins or hBST2 were fixed with 4% paraformaldehyde at 4°C for 20 minutes followed by blocking and permeabilization using 3% bovine serum albumin (BSA) and 0.3% Triton-X for 30 minutes. The cells were then stained with rabbit α-flag and rabbit α-myc antibodies diluted in 10% FBS for 1 hour at room temperature. The cells were washed thrice with PBST and incubated in Alexa Fluor^®^ 488-conjugated goat α-rabbit IgG H&L (Abcam) and Alexa Fluor^®^ 647-conjugated goat α-mouse IgG H&L (Abcam) diluted in 10% FBS for 1 hour at room temperature. After a washing step with PBST, cells were mounted with Prolong^TM^ Gold Antifade Mountant with DAPI (Invitrogen). The samples were observed using a Fluoview^TM^ FV1000 confocal microscope (Olympus).

### Western blot analysis

Cleared lysates or culture supernatants were loaded onto SDS-PAGE gels to separate proteins for transfer onto nitrocellulose membranes. The membranes were blocked with 5% skim milk for 1 hour and probed with a primary antibody diluted in 5% skim milk for 1 hour. The primary antibodies include rabbit α-myc (Abcam), mouse α-myc (Invitrogen), rabbit α-hBST2 (Abcam), rabbit α-flag (Abcam) and mouse α-β-actin (Cell signaling) antibodies. After washing the membranes thrice with Tris-buffered saline (25 mM Tris-HCl, 0.15 M NaCl, 0.1% Tween 20, pH 7.2) (TBST), the membranes were probed with HRP-conjugated goat α-mouse or α-rabbit IgG antibodies for 1 hour and then washed with TBST. Protein bands were detected using chemiluminescence substrate (Bio-Rad) and visualized on a Chem-iDoc™ XRS+ imager (Bio-Rad).

### Co-immunoprecipitation assay

HEK293T cells were cotransfected with plasmids expressing RABV G and flag-hBST2 using Fugene^®^ HD transfection reagent. At 48 hpt, cell lysates were harvested using 200 μl of pre-cooled IP lysis buffer (Thermofisher Scientific) supplemented with a protease inhibitor cocktail (Thermofisher Scientific). Cleared lysates were incubated with mouse α-myc agarose (Thermofisher Scientific) with gentle rocking overnight at 4°C. Immunoprecipitates were washed with 500 μl TBST for five times. The samples were then eluted in 60 μl of sample buffer followed by SDS-PAGE and western blot analyses.

### Purification of RABV-G VLPs and transmission electron microscopy (TEM)

HEK293T cells seeded in 15-cm^2^ dishes (8 dishes) were transfected with 18 μg (per dish) of plasmid expressing RABV Gmyc complexed with Fugene^®^ HD transfection reagent (Promega). The cells were maintained in 30 ml of Opti-MEM^TM^ media without FBS at 37°C in a humidified atmosphere at 5% CO2 for 72 hours. The supernatants were collected and pre-cleared by centrifugation at 1,000 × g for 5 minutes. Precleared supernatants were centrifuged at 10,000 × g for 30 minutes to remove cell debris and filtered through 0.45 μm nylon membrane filter (Pall, NY, USA). The supernatants were then concentrated through 50kDa Amicon™ Ultra-5 Centrifugal Filter Units (Merck) by centrifuging at 3,500 × g for 10 minutes. Concentrated supernatants were layered over a 10 ml 30% sucrose cushion in 25 mm × 89 mm PP Thick-walled Tube (Sorval^®^, NC, USA) and centrifuged at 100,000 × g for 3 hours (Surespin^TM^ 630/36 Rotor, Sorvall^®^ WX+ Ultracentrifuge Series, Thermofisher Scientific). The supernatant and sucrose cushion were gently removed by pouring off and pipetting. The pellets were resuspended in 200 μl PBS.

The RABV-G VLPs were negatively stained with 3% phosphotungstic acid (EMS, PA, USA) on a carbon coated copper grid (EMS). Briefly, the purified sample (20 μl) was dropped onto a carbon coated copper grid (EMS) and incubated for 30 minutes. The sample was then removed. The grid was washed twice with PBS (1 min per each round). Phosphotungstic acid was then added onto the grid and incubated for 2 minutes and removed. The grid was dried in a vacuum incubator before images were taken using TEM (HITACHI H7700, Japan) at 80KV.

### RABV-VLP release assay

HEK293T cells seeded in 10-cm^2^ dishes were transfected with 6 μg of plasmid expressing RABV Gmyc and flaghBST2 complexed with Fugene^®^ HD transfection reagent (Promega). The cells were maintained in 10 ml of Opti-MEM^TM^ media without FBS at 37°C in a humidified atmosphere at 5% CO2 for 72 hours. The supernatants were collected and pre-cleared by centrifugation at 1,000 × g for 5 minutes. Precleared supernatants were centrifuged at 10,000 × g for 30 minutes to remove cell debris and filtered through 0.45 μm nylon membrane filter (Pall). The supernatants were then concentrated through 50kDa Amicon™ Ultra-5 Centrifugal Filter Units (Merck) by centrifuging at 3,500 × g for 10 minutes. Concentrated supernatants were layered over a 5 ml 30% sucrose cushion in 14 mm × 89 mm PA thin-walled tube (Thermofisher Scientific) and centrifuged at 85,000 × g for 3 hours (TH-641 Rotor, Sorvall® WX+ Ultracentrifuge Series, Thermofisher Scientific). The supernatant and sucrose cushion were gently removed by pouring off and pipetting. The pellets were resuspended in 200 μl PBS. The samples were subjected to a western blot analysis to examine RABV G expression.

### Statistical analysis

The differences in mean values between two groups or more were analyzed using the one-way ANOVA methods, respectively, using GraphPad Prism 5.0 (GraphPad Software Inc.). *p* values of < 0.05 were considered statistically significant. All data are presented as means ± standard deviation (SD) of triplicates, unless stated otherwise.

## Results

### Overexpressed hBST2 decreases RABV release

**Fig 1:**
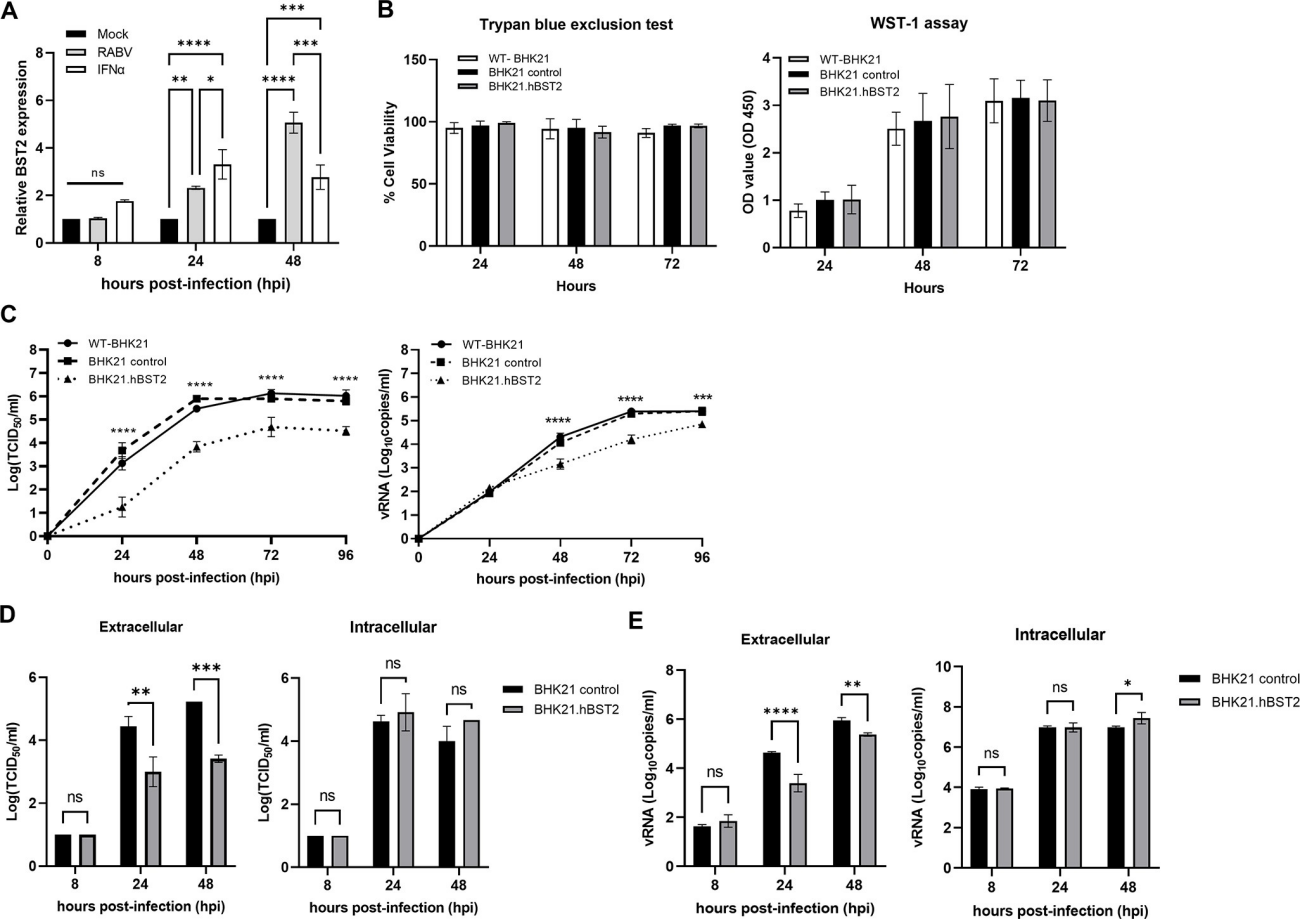
The effects of BST2 on RABV replication. (A) BHK21 cells were infected with RABV and collected for BST2 mRNA measurement at the indicated time points. Cells treated with IFN-α were used as a positive control. (B) BHK21 cells including wild-type BHK21.C13 (WT-BHK21), BHK21 stably expressing hBST2 (BHK21.hBST2) and BHK21 control (transduced with control lentivirus) were harvested and subjected to trypan blue exclusion test and WST-1 assays to determine cell viability. (C) WT-BHK.21, BHK21 control and BHK21.hBST2 were infected with RABV at an MOI of 0.05. Supernatants were harvested for TCID50 virus titration and RT-qPCR of the vRNA. (D-E) hBST2-BHK21 or BHK21 cells were infected with RABV at an MOI of 1. To determine virus titers in the extracellular and intracellular compartments, supernatants and cell lysates were subjected to (D) virus titration (TCID50) and (E) vRNA quantification. Error bars represent means ± SD of triplicate samples. Statistical significances were calculated by one-way ANOVA. ns, no statistical difference; *, *p*<0.05; **, *p*<0.01; ***, *p*<0.001; ****, *p*<0.0001.

Type I IFN production can be triggered by RABV infection and then turns on ISGs [29, 30], but BST2 induction during RABV infection has not been reported. As a result, we investigated whether BST2 expression is increased during RABV infection. Using a permissive cell line as a model, BHK21.C13 cells were infected with RABV or treated with IFN-α as a positive control. RT-qPCR was used to determine relative mRNA expression of BST2 at 8, 24, and 48 hpi. RABV infection induced BST2 expression at 24 and 48 hpi, as shown in Fig 1A. hBST2-expressing BHK21 cells (BHK21.hBST2) were generated to examine whether BST2 could inhibit RABV production. The expression level of hBST2 in BHK21.hBST2 was confirmed by western blot analysis (S1 Fig). Cell viability of wild-type BHK21.C13 cells (WT-BHK21), mock transduced BHK21.C13 cells (BHK21 control), and BHK21.hBST2 cells was measured, confirming that the cells proliferated and had metabolic activity at similar levels (Fig 1B). WT-BHK21, BHK21 control and BHK21.hBST2 cells were infected with a Thai-strain RABV virus at an MOI of 0.05. The supernatants were collected at various time points for virus titration. The results showed that RABV infectious titers and viral RNA levels recovered from the supernatants of infected BHK21.hBST2 cells were significantly lower than that of the control cells (Fig 1C). Virus replication of another RABV strain, CVS-11, was also restricted in BHK21.hBST2 cells (S2 Fig).

After infecting WT-BHK21, BHK21 control and BHK21.hBST2 cells with RABV at an MOI of 1, cell lysates and supernatants were harvested at 8, 24, and 48 hpi for virus titration and vRNA measurement. At 24 and 48 hpi, a significant increase in virus titers (TCID50/ml) and vRNAs was observed in WT-BHK21 and BHK21 control cells compared to BHK21.hBST2 cells, presumably from RABV particles released into the supernatant. However, there was no statistical difference in viral titers and vRNA in cell lysates, particularly at 8 and 24 hpi (Fig 1D and 1E). These results indicate that hBST2 impedes RABV growth kinetics while having no effect on intracellular RABV reproduction, especially at early time points. We thus hypothesized that hBST2 probably interferes with RABV replication by limiting virus budding as seen with VSV [24].

**Fig 2:**
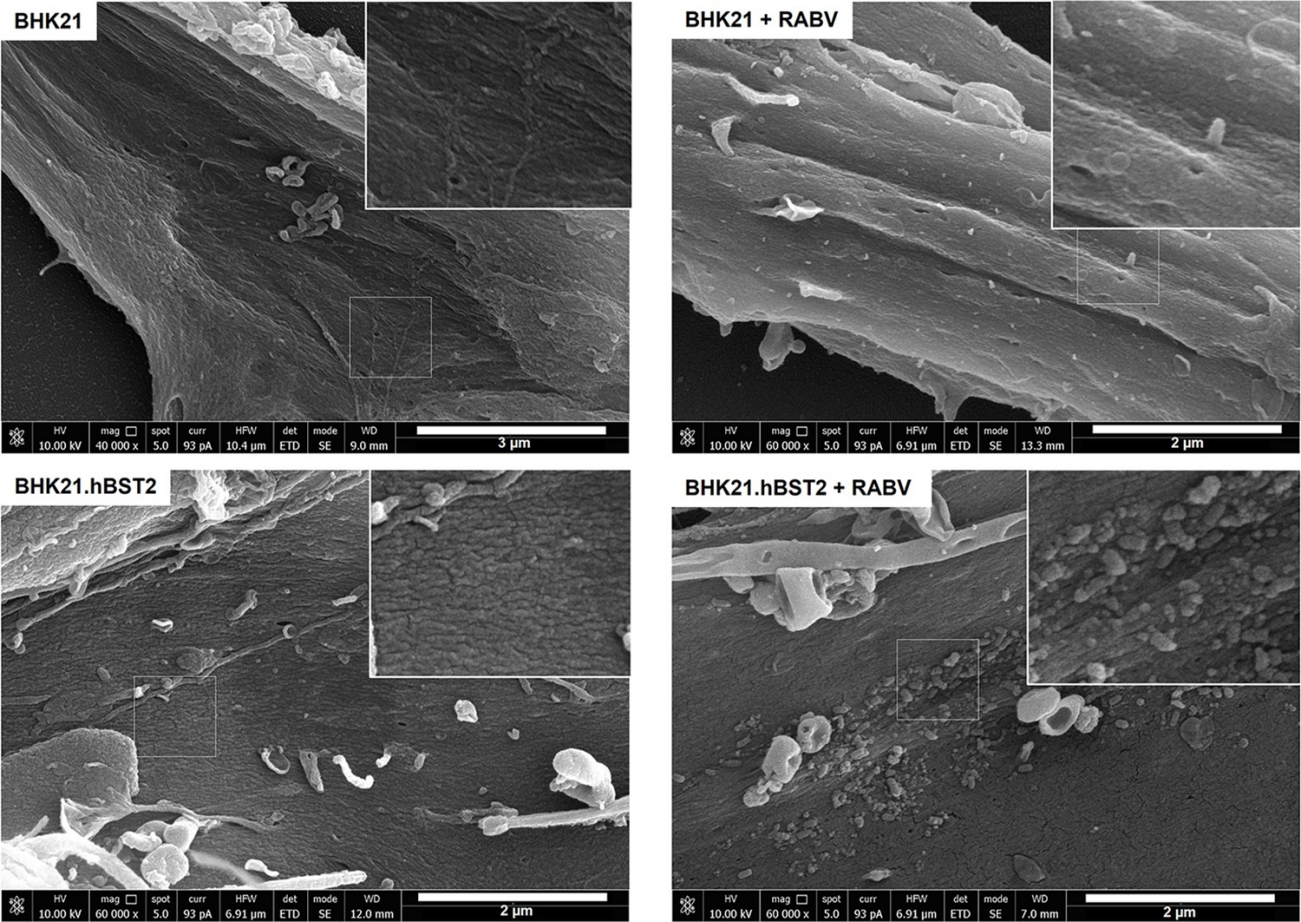
hBST2 restricts RABV particles to the surface of infected cells. hBST2-BHK21 cells or BHK21 control cells were infected with RABV at an MOI of 1. At 24 hpi, cells were fixed and prepared for SEM. SEM was performed with a field emission scanning electron microscope (Versa 3D, FEI), operated at 10 kV.

One of the primary ways that BST2 can combat viral infections is by tethering viral particles to the cell surface. To investigate whether hBST2 has a similar effect on RABV particles, we utilized scanning electron microscopy (SEM) to visualize RABV particles on infected BHK21.hBST2 cells. As depicted in Fig 2, an abundance of RABV particles was detected on the cell surface of BHK21.hBST2, while there was only a scarce number of RABV particles on the BHK21 control cells. These results suggest that hBST2 limits the release of RABV particles, thereby impeding viral replication.

### RABV G alters hBST2 localization and modulates hBST2 modification

Throughout the BST2 expression process, the protein is transported intracellularly via various mechanisms through different host cell organelles. After being translated, BST2 undergoes post-translational modifications such as N-glycosylation and disulfide bond formation in the ER. It is then directed to the Golgi complex/TGN and transported to the apical region of polarized epithelial cells [31]. These processes are often exploited by viruses to enhance viral replication and evade antiviral defenses. Therefore, it is possible that RABV proteins interact with BST2 and/or alter BST2 expression, as has been observed with other viruses [32].

**Fig 3:**
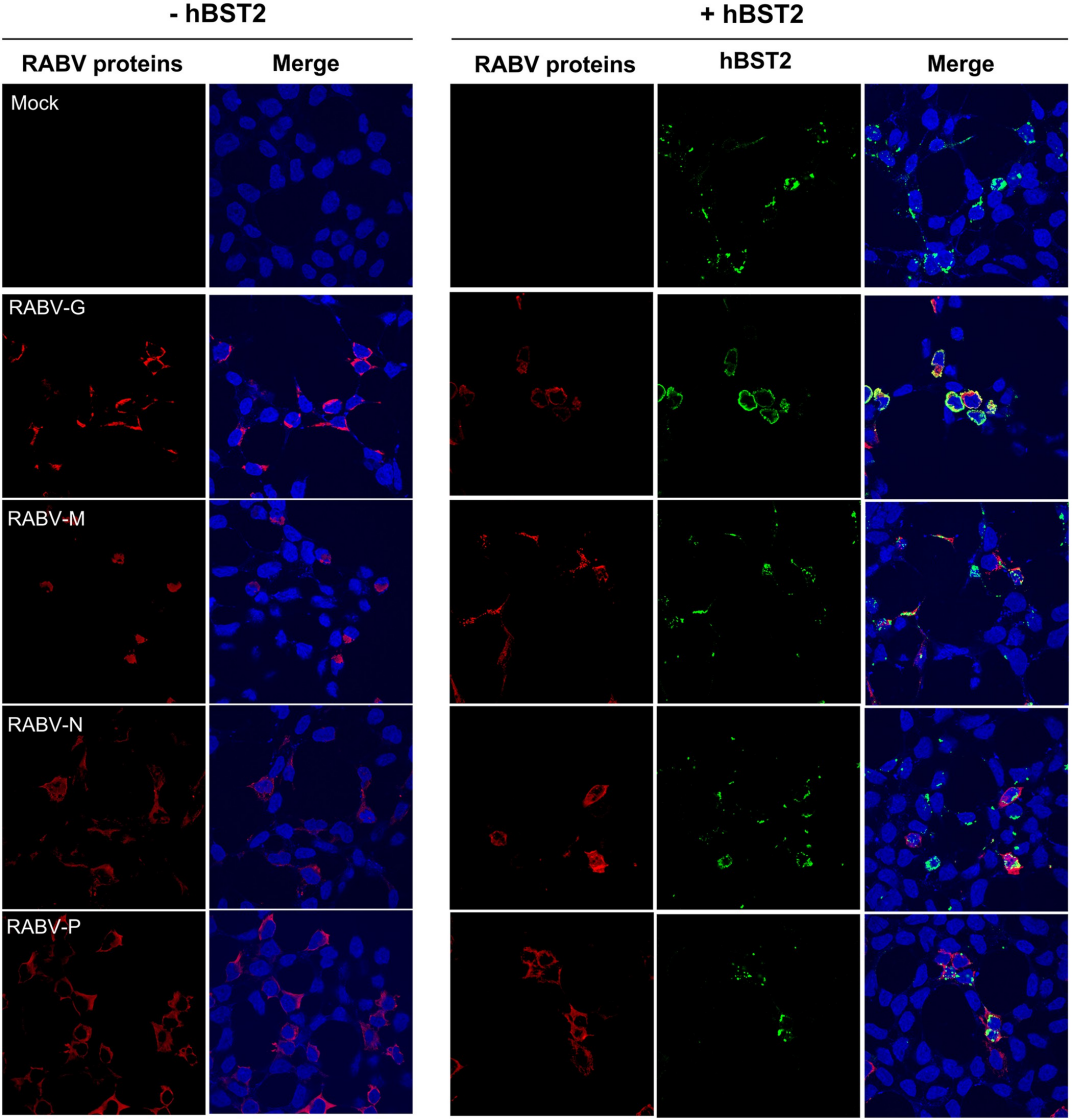
hBST2 translocation to the cell surface is induced by RABV G protein. HEK293T cells were transfected with plasmids expressing individual myc-tagged RABV proteins and flag-tagged hBST2. At 24 hpt, cells were fixed with 4% paraformaldehyde and probed with mouse α-myc and rabbit α-flag primary antibodies to detect RABV proteins and hBST2, respectively, followed by Alexa Fluor^®^ 488-conjugated goat α-rabbit IgG H&L and Alexa Fluor^®^ 647-conjugated goat α-mouse IgG H&L secondary antibodies. Localization of the proteins was visualized by confocal microscopy.

Using confocal microscopy, we aimed to investigate whether RABV proteins, namely RABV G, M, N, and P proteins, influenced hBST2 localization and expression in transfected cells. As illustrated in Fig 3, hBST2 was primarily localized to the plasma membrane in cells transiently producing RABV G, whereas in the absence of RABV G, it formed a punctate pattern in the cytoplasm. No significant changes in hBST2 localization were observed when RABV M, N, and P were co-expressed (Fig 3). The remarkable translocation of hBST2 to the cell surface induced by RABV G might be associated with its ability to inhibit virus particle budding.

**Fig 4:**
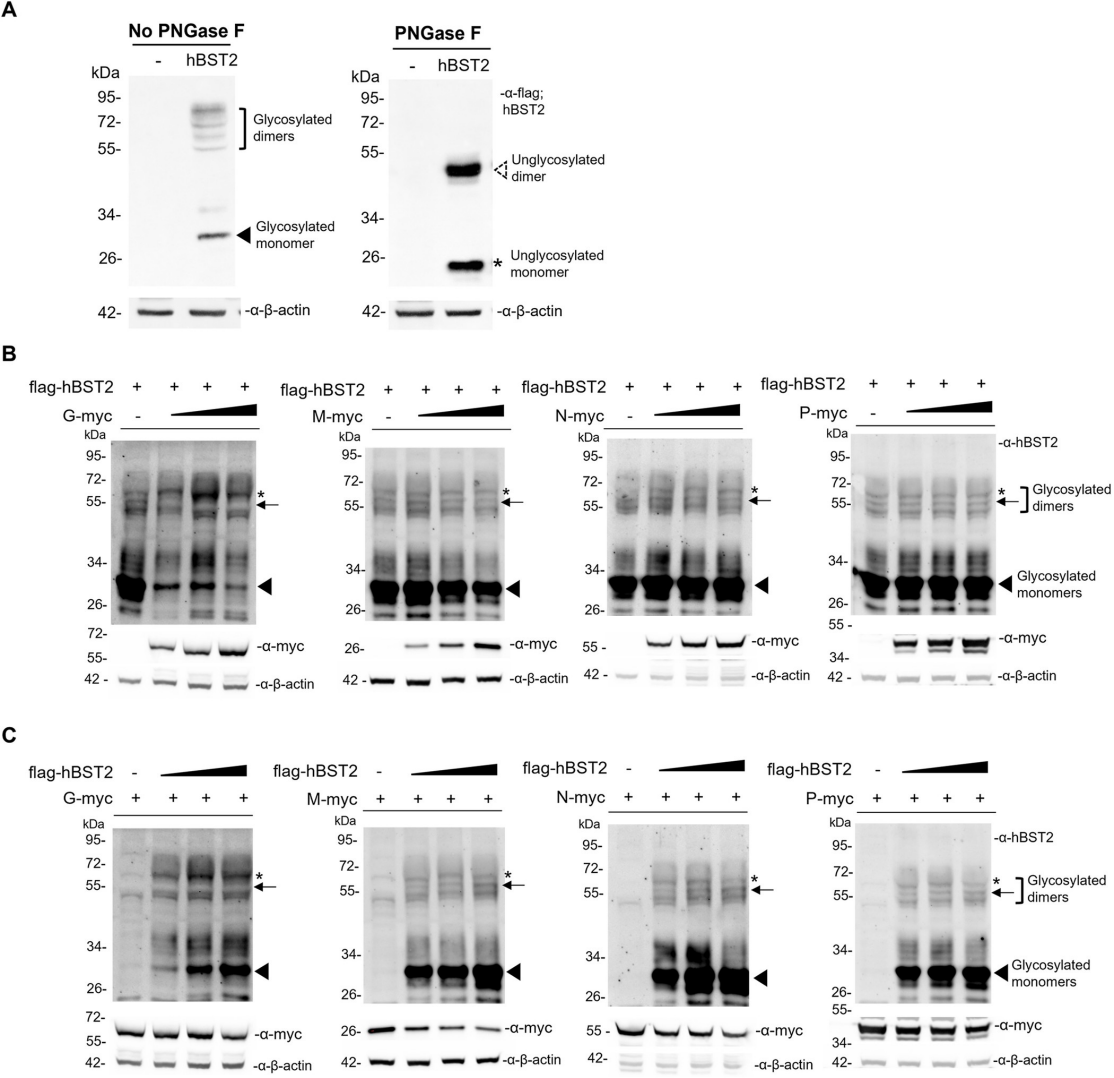
hBST2 and RABV protein expression in transfected cells expressing hBST2 and RABV proteins. (A) HEK293T cells were transfected with plasmids expressing flag-hBST2. Cell lysates were prepared and incubated in the presence or absence of PNGase F and subjected to western blot analysis of hBST2. HEK293T cells were transfected with (B) 1 μg of flag-hBST2 plasmid and an increasing amount (0.5, 1 and 2 μg) of plasmids encoding RABV G-myc, M-myc, N-myc and P-myc or (C) 1 μg of plasmids encoding individual myc-tagged RABV proteins with an increasing amount of flaghBST2 plasmid (0.5, 1 and 2 μg). At 48 hpt, cell lysates were collected and analyzed by western blotting for RABV protein and hBST2 expression using rabbit α-myc and α-human BST2 antibodies, respectively. Arrow heads indicate glycosylated monomeric hBST2 of approximately 30 kDa. Arrows indicate a low molecular weight, glycosylated dimeric hBST2 of approximately 55 kDa. Asterisks indicate high molecular weight glycosylated dimeric hBST2 of approximately 65 kDa.

Since BST2 has been shown to be heavily glycosylated and appears as large smears on a western blot, we thereby examined migration patterns of hBST2 transiently expressed in HEK293T cells. Cell lysates of HEK293T cells transfected with a flag-BST2 plasmid were left untreated or treated with PNGase F to remove all N-linked glycans on the proteins. The results revealed that hBST2 in the PNGase F-untreated sample displayed heterogeneity, with multiple protein bands observed (Fig 4A). The band at approximately 30 kDa was identified as a monomer and immature BST2 due to high-mannose-type carbohydrate modifications in the ER [16, 33]. The multiple higher molecular weight protein bands of approximately 55–72 kDa were predicted to be the dimeric hBST2 with the addition of complex carbohydrates [34]. After the PNGase F treatment, hBST2 was detected as two discrete protein bands of approximately 20 and 40 kDa, both lower than their corresponding bands of glycosylated monomeric and dimeric hBST2 bands in the untreated sample This confirms that the bands observed in the untreated cell lysates are the glycosylated monomeric and dimeric forms of hBST2, respectively.

To investigate if the presence of RABV proteins alters the post-translational modifications of hBST2, we performed western blot analysis of lysates from HEK293T cells co-transfected with hBST2 and individual RABV genes. Our results showed that expression levels of the higher molecular weight form of dimeric hBST2 (approximately 65 kDa) increased, while the lower molecular weight form (approximately 55 kDa) levels decreased in the presence of RABV G in a dose-dependent manner. Additionally, the 30 kDa band, representing the mannose-glycosylated monomeric form of hBST2, was significantly reduced in the presence of RABV G (Fig 4B). However, no significant differences in the expression of any hBST2 forms were observed in the presence of RABV P, M, or N (Fig 4B). These findings indicate that RABV G induces changes in post-translational modifications, such as glycosylation or dimerization [16]. We also examined whether hBST2 can downregulate viral protein expression to restrict viral replication. As shown in Fig 4C, increasing levels of hBST2 did not significantly alter the expression levels of RABV G, N, and P, while slightly decreasing that of RABV M (Fig 4C). Taken together, our results suggest that RABV G protein drives hBST2 modifications, leading to increased levels of the high molecular weight form and modulating hBST2 translocation to the cell surface (Fig 3).

### hBST2 inhibits RABV G-containing VLPs (RABV-G VLPs)

**Fig 5:**
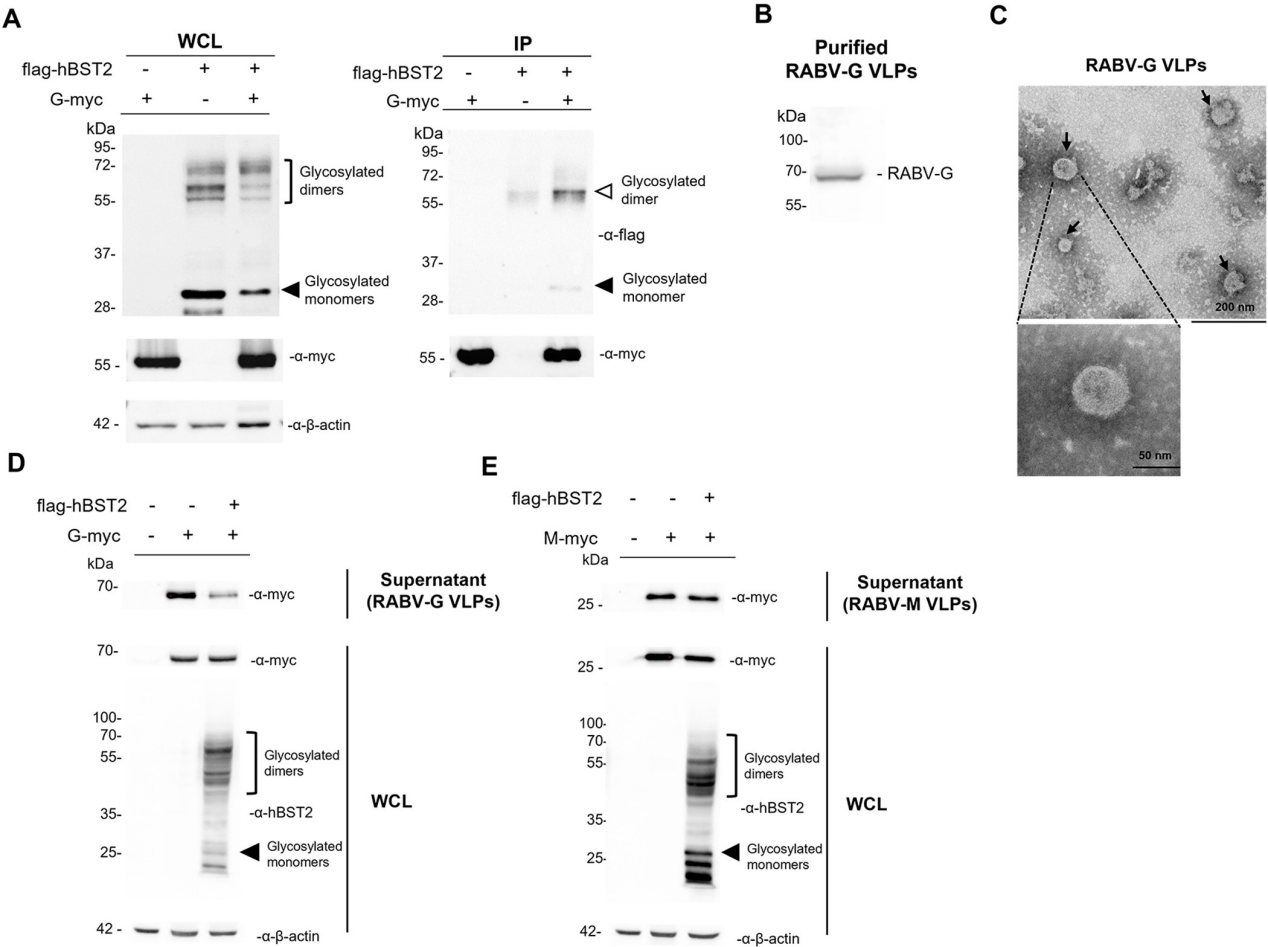
hBST2 interacts with RABV G and restricts RABV-G VLP release. (A) HEK293T cells were transfected with plasmids expressing flag-hBST2 and RABV G-myc. At 48 hpt, cell lysates were collected and immunoprecipitatated using mouse α-myc beads. Eluted proteins were probed with rabbit α-myc and α-flag antibodies to examine levels of RABV G and hBST2 using rabbit α-myc and α-flag antibodies, respectively. (B-C) RABV- VLPs purification and VLP release assay. HEK293T cells were transfected with plasmids expressing RABV G-myc. At 72 hpt, cell supernatant was harvested, precleared and subsequently purified by sucrose cushion ultracentrifugation. (B) RABV G expression in the purified sample was examined by western analysis. The membrane was probed with rabbit α-myc to detect RABV G. (C) The purified RABV-G VLPs were negative staining with phosphotungstic acid on carbon-coated copper grid for transmission electron microscopy (TEM). Scale bars are 200 nm (Upper) and 50 nm (Lower). (D-E) VLP release assay was performed to measure RABV VLPs in the presence of hBST2 expression. HEK293T cells were transfected with plasmids expressing (D) RABV G-myc or (E) RABV M-myc alone or together with flag-hBST2 plasmid. At 72 hpt, cell supernatants and pellets were harvested to measure the presence of RABV-G. Supernatants were purified through sucrose cushion ultracentrifugation to collect RABV-G VLPs. The samples were subjected to western blot analysis using rabbit α-myc and α-flag antibodies. WCL; whole cell lysate.

We next investigated whether hBST2 physically interacts with the RABV envelope glycoprotein. Plasmids expressing hBST2 and RABV G were co-transfected into HEK293T cells. Cells were lysed 48 hours post-transfection and then co-immunoprecipitated with mouse α-myc beads. Western blotting revealed that RABV G physically binds to both glycosylated monomeric and dimeric forms of hBST2 (Fig 5A). RABV G has an intrinsic force and autonomous exocytosis activity contributing to virus budding [35]. RABV G alone has been demonstrated to induce a formation of RABV virus-like particles (VLPs) in HEK293 cells [36, 37]. To this end, we further examined the ability of hBST2 to restrict the release of RABV G-based VLPs (RABV-G VLPs). We initially confirmed that the RABV-G VLPs were produced and released from the G-transfected HEK293 cells. The supernatant was collected and subjected to VLP purification using sucrose cushion ultracentrifugation as previously described [36, 37] with slight modifications. RABV-G was detected by western blot (Fig 5B) and the presence of purified RABV-G VLPs was visualized by TEM (Fig 5C). The RABV-G VLPs were observed as spherical particles with the presentation of G proteins on the lipid surface (Fig 5C). The size of the VLPs ranged from 50 to 60 nm.

We next determined the effect of hBST2 in restricting RABV-G VLP release using the RABV VLP release assay. RABV-G VLPs were produced from cells transfected with the RABV G plasmid alone or in combination with the hBST2 plasmid. The VLPs were purified using sucrose cushion ultracentrifugation. Despite similar levels of intracellular G expression, the amount of RABV G protein in the supernatant obtained from cells co-expressing hBST2 and RABV G was lower than that of cells expressing RABV G alone (Fig 5D), suggesting that hBST2 restricts the release of RABV-G VLPs. Several studies have shown that the BST2 retains progeny virions on the cell surface by tethering the viral and cellular membranes. We investigated whether hBST2-mediated RABV-VLP tethering was specific for RABV-G VLPs by testing if it inhibited VLPs formed by a different RABV protein. Previous studies have demonstrated that the M protein of rhabdovirus alone can generate VLPs [38, 39] and is vital for virus assembly and budding [40]. Therefore, we produced RABV M-based VLPs and conducted a VLP release assay. The results showed that hBST2 did not inhibit RABV-M VLP budding (Fig 5E), indicating that the restriction of VLP release was specific to RABV G-containing particles. This could be because both proteins are predicted to be lipid raft proteins [13, 41]. RABV M, however, was not found to be co-precipitated with hBST2 (S3 Fig), possibly because RABV M-VLPs are physically separated from hBST2-containing lipid rafts and therefore their release is not inhibited by hBST2.

### hBST2 dimerization and glycosylation are dispensable for RABV-G VLP restriction and regulation of RABV infection

**Fig 6:**
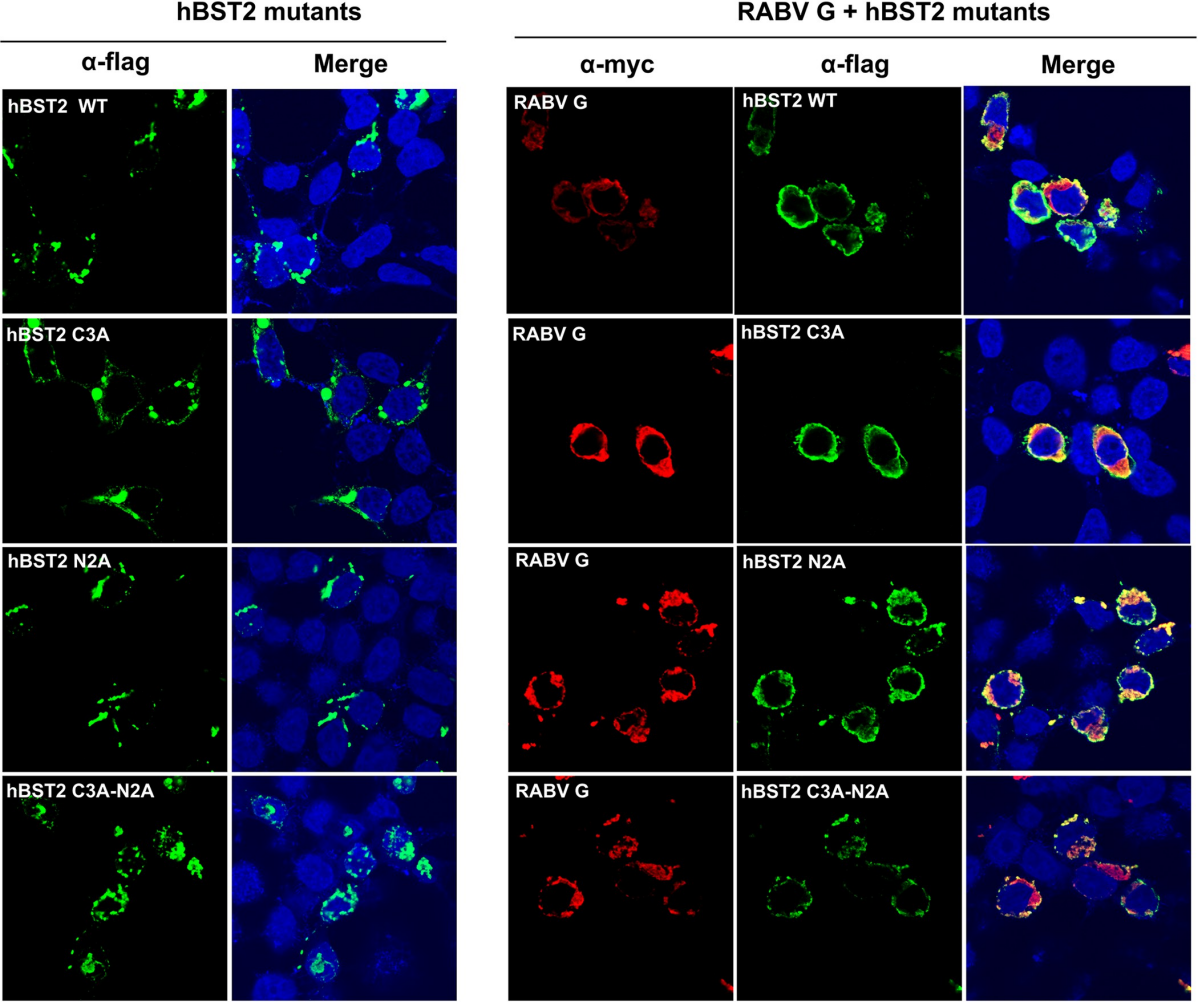
hBST2 mutants with dimerization and glycosylation defects colocalized with RABV G. HEK293T cells were transfected with plasmids expressing RABV Gmyc and flaghBST2. At 24 hpt, cells were fixed and probed with mouse α-myc and rabbit α-human BST2 primary antibodies to detect RABV G and hBST2, respectively followed by Alexa Fluor^®^ 488-conjugated goat α-rabbit IgG H&L and Alexa Fluor^®^ 647-conjugated goat α-mouse IgG H&L secondary antibodies. Localization of the proteins were visualized by confocal microscopy.

As the presence of RABV G increased the amount of high molecular weight hBST2, we next examined whether dimerization and N-glycosylation of hBST2 are necessary for the inhibition of RABV release. First, we constructed plasmids encoding hBST2 with mutations at the conserved cysteine positions 53, 63 and 91 (C3A) that involve in the formation of disulfide-linked dimers, mutations at asparagine positions 65 and 92 (N2A), which disrupt potential sites for N-linked glycosylation [16], and C3AN2A mutations. These plasmids were used to cotransfect HEK293T cells alone or together with a plasmid encoding RABV G. At 24 hpt, confocal microscopy was performed to examine protein localization. As shown in Fig 6, it was found that wild-type and all BST2 mutants appeared in punctate patterns in the absence of RABV G, but became more dispersed around the cells in the presence of RABV G. More importantly, both wild-type and all the mutants appeared to colocalize with the RABV G (Fig 6) and a proportion of N2A and C3AN2A mutants retained a puncta-like distribution in the cytoplasm due to mutation of glycosylation sites (N2A) [42, 43]. Colocalization was not seen when hBST2 was co-expressed with other RABV proteins (M, N, and P) (S4 and S5 Figs).

**Fig 7:**
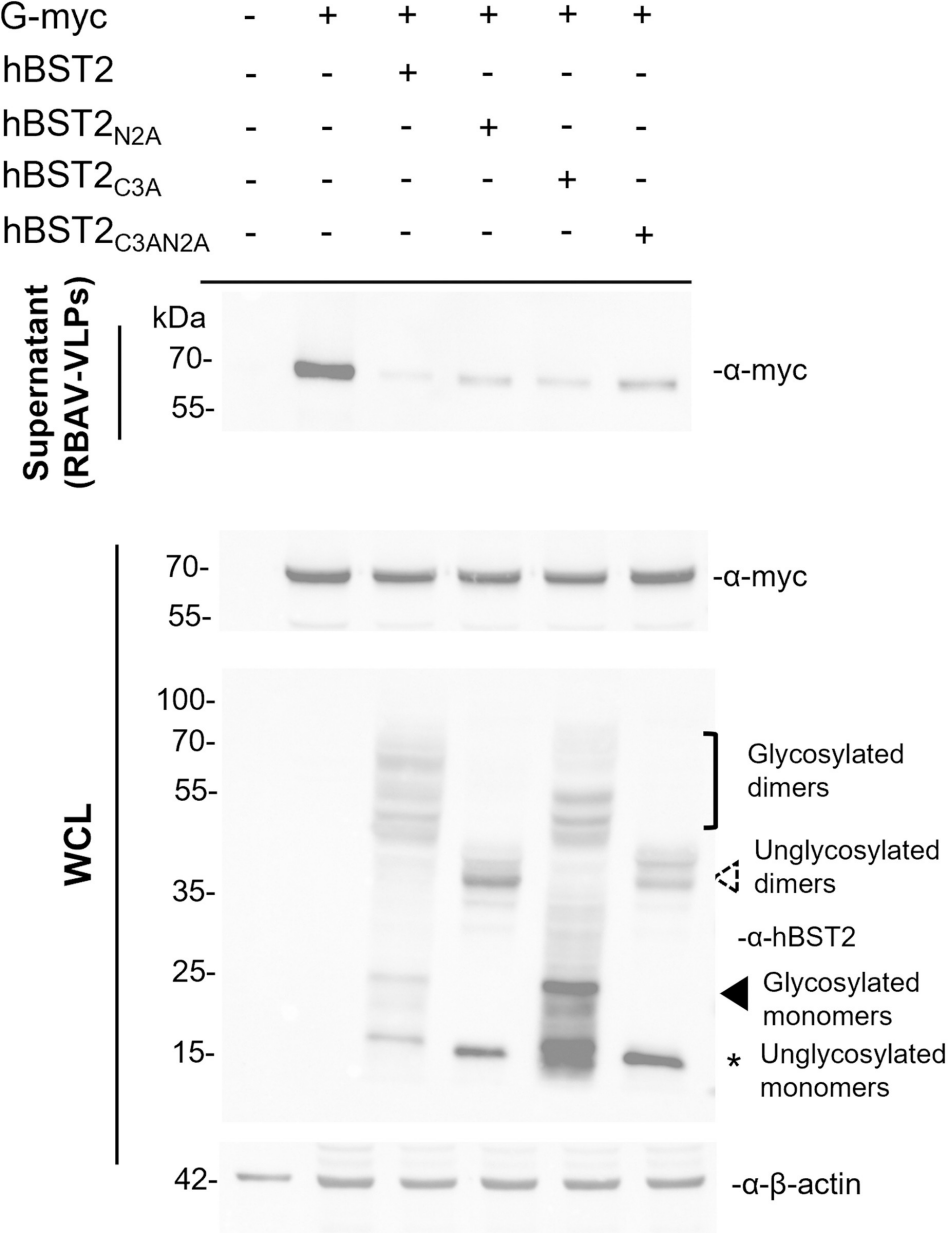
Restriction of RABV-VLP release by dimerization/glycosylation-defective hBST2. HEK293T cells were transfected with plasmids expressing RABV G and wild-type or mutated hBST2. At 72 hpt, cell supernatants and pellets were harvested to examine RABV-VLP release and levels of intracellular RABV G and hBST2 expression, respectively. Supernatants were purified through sucrose cushion ultracentrifugation to collect RABV-G VLPs. The samples were subjected to western blot analysis to examine RABV G and hBST2 levels using rabbit α-myc and α-human BST2 antibodies. WCL; whole cell lysate.

To examine whether these BST2 mutants can inhibit RABV release, a RABV-VLP release assay was performed. HEK293T cells were transfected with plasmids encoding wild-type or mutated hBST2 alone or together with a plasmid encoding RABV G. At 72 hpt, supernatants and cell lysates were harvested for western blot analysis. As shown in Fig 7 it was found that in the presence of all hBST2 forms, wild-type and the mutants, the amounts of RABV VLP found in the supernatants were significantly lower than that of cells transfected with RABV G alone. Particularly, the intracellular level of RABV G was unaltered regardless of the presence of any form of hBST2. Altogether, our data suggest that hBST2 colocalizes with RABV G and restricts RABV release regardless of its dimerization and glycosylation states. It is important to note that the N2A and C3AN2A mutants were expressed at relatively lower levels compared to other constructs. It is plausible that these mutants were partially degraded through the lysosomal pathway due to post-translational mis-trafficking resulting from glycosylation mutations as previously described [42].

**Fig 8:**
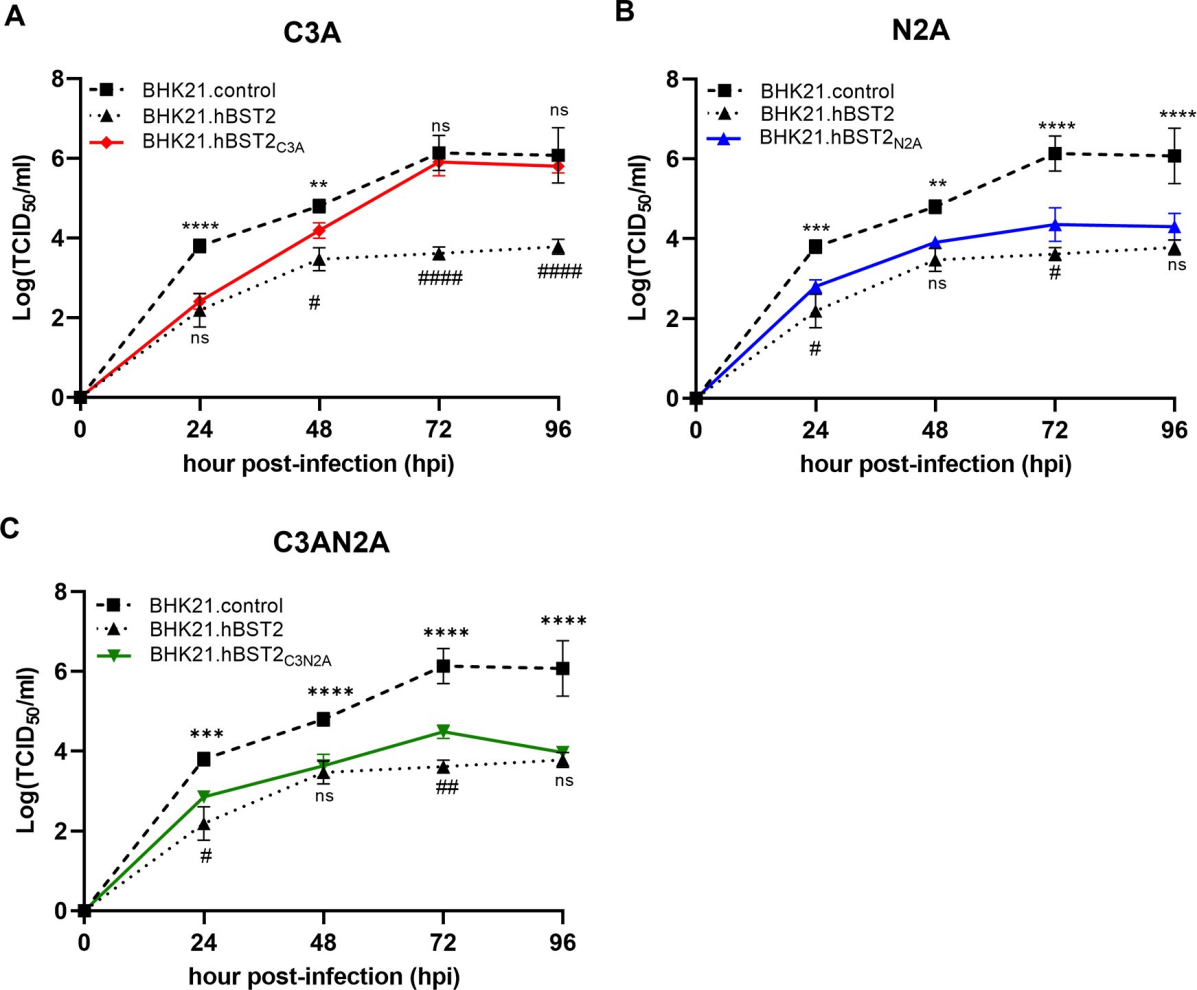
RABV replication kinetics in BHK21 stably expressing dimerization and glycosylation defective hBST2. BHK21 carrying hBST2 mutants (C3A, N2A and C3AN2A) or BHK21.hBST2 and BHK21 control (transduced with a control lentivirus) were infected with Thai-strain RABV at an MOI of 0.05. The supernatants were harvested at indicated time points for TCID50 assay. Error bars represent means ± SD of independent experiments. Statistical significances were calculated by one-way ANOVA. **, *p*<0.01; ***, *p*<0.001; ****, *p*<0.0001 and ns, no significant difference. Noted that (*) indicates significant differences of virus titers between BHK21 carrying hBST2 mutants and BHK21 control and (#) indicate significant differences of virus titers between BHK21 carrying hBST2 mutants and BHK21.hBST2.

Finally, we established BHK21 cell lines that expressed hBST2 mutants with defects in dimerization, glycosylation, or both, and analyzed their effects on RABV replication using growth curve analysis. The results showed that all hBST2 mutants inhibited RABV replication, but they were not as effective as the wild-type hBST2 in reducing RABV infectious titer (Fig 8A–8C). Interestingly, RABV titers in cells expressing the dimerization-defective hBST2 (C3A) were reduced at early time points (24 and 48 hpi) but increased to levels similar to the BHK21 control at late time points (72 and 96 hpi) (Fig 8A). In contrast, the glycosylation-defective hBST2 (N2A) and the double mutation hBST2 (C3AN2A) suppressed RABV replication at all time points (Fig 8B and 8C). These findings show that hBST2 restriction of RABV release is unaffected by glycosylation while RABV-G VLP release is unaffected by both glycosylation and dimerization. Altogether, these results suggest that hBST2 may modulate RABV infection through multiple mechanisms, similar to other viruses, and that RABV may possess viral proteins that counteract hBST2 functions.

## Discussion

BST2 is a host antiviral protein that has gained much interest for its roles in virus-host interaction. Resembling a dimeric bridge, BST2 is a membrane protein composed of an N-terminal transmembrane domain, a coiled-coil ectodomain, and a C-terminal GPI anchor. BST2’s unique structure enables it to anchor virions to the host cell surface [15, 44–46]. Apart from this, BST2 also inhibits viral replication by suppressing viral protein expression and shuttling viral proteins towards degradation [47, 48]. While BST2’s antiviral effects against many viruses such as HIV, influenza A, and human cytomegalovirus are well established [49–51], its impact on RABV infection is not clearly defined. To address this, we investigated the antiviral effects of hBST2 on RABV infection at different stages of the viral cycle. Our findings indicated that hBST2 has no effect on intracellular infectious viral titers, but it does significantly suppress the release of RABV virions. This suppression is evident by the decrease in virus titers in the supernatant and the accumulation of RABV particles on the cell surface of a cell line expressing hBST2. Upon further examination, it appears that RABV G may be involved in hBST2’s antiviral activities, as indicated by binding of hBST2 and RABV G (Fig 5A) and the specific restriction of RABV G-VLP release (Fig 5D). Furthermore, the presence of RABV G induces the translocation of hBST2 to the cell surface (Fig 3). In contrast to the findings of our study, existing reports highlight the antagonistic effects of viral glycoproteins that engage in physical interactions with BST2. For example, Ebola GP and HIV-2 Env have been shown to remove BST2 from the cell surface, thereby facilitating virion release [22, 52]. Additionally, BST2 has been shown to promote the entry of human cytomegalovirus [50]. Future studies dissecting the functional anatomy of RABV G with respect to its trafficking and interaction with hBST2 would be critical to elucidating the mechanism for the observed G-specific restriction and whether direct G-hBST2 interaction is a requisite for such restriction.

The dimeric bridge-like structure of BST2 has generally been shown to be essential for its antiviral function, as it enables BST2 to tether virions to the cell surface and prevent their release [16]. This has been demonstrated in various studies, including those on HIV-1 [16, 34], SARS-CoV [53], and SARS-CoV-2 [54]. The effect of post-translational modifications, such as glycosylation, on BST2’s antiviral activity has also been investigated, with some studies suggesting that BST2’s glycosylation is important for its antiviral function [42, 55]. On the other hand, studies on Lassa and Marburg viruses showed that the antiviral functions of BST2 do not require dimerization [21], indicating that BST2 can suppress viral replication through alternate virus-specific mechanisms. In this study, we explored whether the dimerization and glycosylation of hBST2 influence RABV replication. We found that BST2 mutants defective in glycosylation, dimerization, or both, were able to inhibit RABV-VLP release similarly to the fully matured glycosylated and dimerized BST2 (Fig 7). These data suggest that the post-translational modifications of BST2 are dispensable for the inhibition of RABV virion release. Our findings also show that all mutants colocalized with RABV-G, implying that hBST2, in addition to binding to RABV G and tethering RABV to the cell surface, may also interact with RABV G intracellularly and inhibit viral release via other unidentified mechanisms.

The ectodomain and C-terminal GPI anchor are required for BST2 to bind directly to viral glycoproteins such as those of HIV-2 [56] and Ebola [22]. Furthermore, BST2 lacking the TM or GPI anchor has been shown incapable of restricting retrovirus release [33, 57]. By constructing hBST2 with deletions in the N-terminal cytoplasmic region (ΔTM), GPI anchor (ΔGPI) and ΔTM-GPI, we intended to identify the hBST2 domain(s) responsible for the interaction of hBST2 with RABV G. Unfortunately, hBST2 truncations, especially the ΔGPI and ΔTM-GPI constructs, yielded extremely low levels of protein expression when co-expressed with RABV-G (S6 Fig) and were insufficient for a protein-protein binding assay. The roles of each hBST2 region in the binding to RABV G remain to be investigated.

As shown for HIV-1 Vpu, Kaposi’s sarcoma-associated herpesvirus (KSHV) K5 and simian immunodeficiency virus Nef and Ebola virus GP that could counteract the antiviral activity of BST2 *in vitro* and *in vivo* [58]. We speculate that RABV proteins may antagonize hBST2-mediated viral restriction. Since our finding showed that RABV replication was inhibited at early time points of infection but continued propagating efficiently in cells stably expressing hBST2 at later time points, hBST2 effects on RABV replication and release are possibly dependent on type I IFN expression activated following virus infection at early time points [59].

Our study employed the BHK21 cell line to investigate the impact of hBST2 on RABV. Although these cells are not the primary target cells of RABV, their ease of manipulation and ability to efficiently support RABV replication has enabled us to gain insight into the mechanisms through which hBST2 suppresses RABV. The finding indicates that RABV infection induced hBST2 expression is consistent with studies by others, which have shown that BST2 expression was induced in primary mouse neurons by a neurotropic measles virus (MV) infection, although BST2 had no effect on viral neuropathogenesis after MV challenge in BST2 KO mice [60]. Therefore, we are presently using our recently developed human-induced pluripotent stem cell (hiPSC)-derived neural cells [27] to examine the roles of BST2 in RABV infection in neurons. To transduce hiPSC-derived neurons, we constructed an adeno-associated virus (AAV) vector that expresses hBST2 driven by the human synapsin 1 gene (hSyn1) promoter. Although we were able to transiently generate hiPSC-derived neurons that expressed hBST2 successfully (S7 Fig), the overexpression of hBST2 in neurons caused cytotoxicity after multiple attempts. As a consequence, we were unable to analyze RABV growth kinetics in hBST2-overexpressing neurons to determine whether hBST2 restricts RABV infection in neurons. Therefore, while this study lays the groundwork for investigating hBST2-RABV interactions, further research is required to establish whether hBST2 can restrict RABV in the nervous system using *in vitro* neuronal models or *in vivo* studies. If similar experiments can be effectively conducted in neurons, this area of research may offer a promising path for novel drug design.

In summary, our data revealed hBST2’s ability to limit RABV-G VLP budding and RABV growth kinetics is independent of its N-linked glycosylation and, in earlier time points, cysteine-linked dimerization. However, whether RABV-G/hBST2 interaction has any direct effect on RABV release and replication requires further investigations. Overall, this research provides novel insights into host antiviral mechanisms that govern RABV infection and helps paint a promising road map for future exploration of BST2-RABV interactions.

## Supporting information

S1 Fig Expression of hBST2 in BHK21 cells. BHK21 cells including wild-type BHK21.C13 (BHK21.WT), BHK21 stably expressing hBST2 (BHK21.hBST2) and BHK21 control (transduced with control lentivirus) were harvested and subjected to western blot analysis. The membrane was probed with rabbit α-human BST2 antibody to detect hBST2 expression.
(TIF)

S2 Fig The effects of hBST2 on RABV CVS11 replication. BHK21 stably expressing hBST2 (BHK21.hBST2) or BHK21 control (transduced with a control lentivirus) were infected with RABV CVS-11 strain at an MOI of 0.05. The supernatants were harvested for TCID50 assay. Error bars represent means ± SD of triplicates. Statistical significances were calculated by one-way ANOVA. **, *p*<0.01; ***, *p*<0.001; ****, *p*<0.0001.
(TIF)

S3 Fig Assessment of hBST2 and RABV M interaction by co-immunoprecipitation. HEK293T cells were transfected with plasmids expressing flag-hBST2 and RABV M-myc. At 48 hpt, cell lysates were collected and immunoprecipitatated using mouse α-myc beads. Eluted proteins were probed with rabbit α-myc and α-flag antibodies to examine levels of RABV M and hBST2 using rabbit α-myc and α-flag antibodies, respectively.
(TIF)

S4 Fig Localization of hBST2 C3A and N2A mutants and RABV proteins. HEK293T cells were transfected with plasmids expressing individual myc-tagged RABV proteins and flag-tagged hBST2 C3A or N2A. At 24 hpt, cells were fixed and probed with mouse α-myc and rabbit α-flag primary antibodies to detect RABV proteins and hBST2 C3A or N2A, respectively, followed by Alexa Fluor^®^ 488-conjugated goat α-rabbit IgG H&L and Alexa Fluor^®^ 647-conjugated goat α-mouse IgG H&L secondary antibodies. Localization of the proteins was visualized by confocal microscopy.
(TIF)

S5 Fig Localization of hBST2 C3AN2A mutant and RABV proteins. HEK293T cells were transfected with plasmids expressing individual myc-tagged RABV proteins and flag-tagged hBST2 C3AN2A. At 24 hpt, cells were fixed and probed with mouse α-myc and rabbit α-flag primary antibodies to detect RABV proteins and hBST2 C3AN2A, respectively, followed by Alexa Fluor^®^ 488-conjugated goat α-rabbit IgG H&L and Alexa Fluor^®^ 647-conjugated goat α-mouse IgG H&L secondary antibodies. Localization of the proteins was visualized by confocal microscopy.
(TIF)

S6 Fig Construction and expression of truncated hBST2. (A) A schematic representing hBST2 truncations include deletion of the N-terminal transmembrane domain (amino acids 1–43: ΔTM), deletion of the GPI anchor (amino acids 161–180: ΔGPI) and both (ΔTM-GPI). (B) HEK293T cells were transfected with the plasmids expressing each truncated hBST2 alone or with RABV-G. At 24 hpt, cell supernatants and pellets were harvested to examine protein expression. The cleared lysates were subjected to western blot analysis to examine hBST2 and RABV G using rabbit α-flag and rabbit α-myc antibodies, respectively.
(TIF)

S7 Fig hIPSC-derived neurons expressing hBST2 transduced by recombinant adeno-associated virus (AAV). To generate recombinant AAV expressing hBST2, transfer vectors pAAV-hSyn1-hBST2-T2A-mcherry (T2A, self-cleaving peptides derived from Thosea asigna virus 2A, was used to cleave the fused hBST2-mcherry protein into two separated peptides) or pAAV-hSyn1-mcherry were constructed and co-transfected with a packaging plasmid (pAAV2/9n addgene: Plasmid #112865) and helper plasmid (pAdDeltaF6, addgene: Plasmid #112867) into HEK293A cells using PEI transfection. Transfected cells were maintained in OptiMEM^TM^ media without FBS for 96 hours. Recombinant AAV in the supernatant was then rescued and purified through an Amicon^®^ Ultra-15 Centrifugal Filter Unit. Concentrated virus was tittered by qPCR (genome copies/μl). hIPSC-derived neurons were transduced with each recombinant AAV at 10^4^ genome copies for 5 days. The representative pictures present transduced neurons at 48 hours post transduction.
(TIF)
