# Supplementary figures and images for: Human BST2 inhibits rabies virus release independently of cysteine-linked dimerization and asparagine-linked glycosylation
Source: PLoS One. 2023 Nov 3;18(11):e0292833. doi: 10.1371/journal.pone.0292833 (PMC10624315; doi:10.1371/journal.pone.0292833)

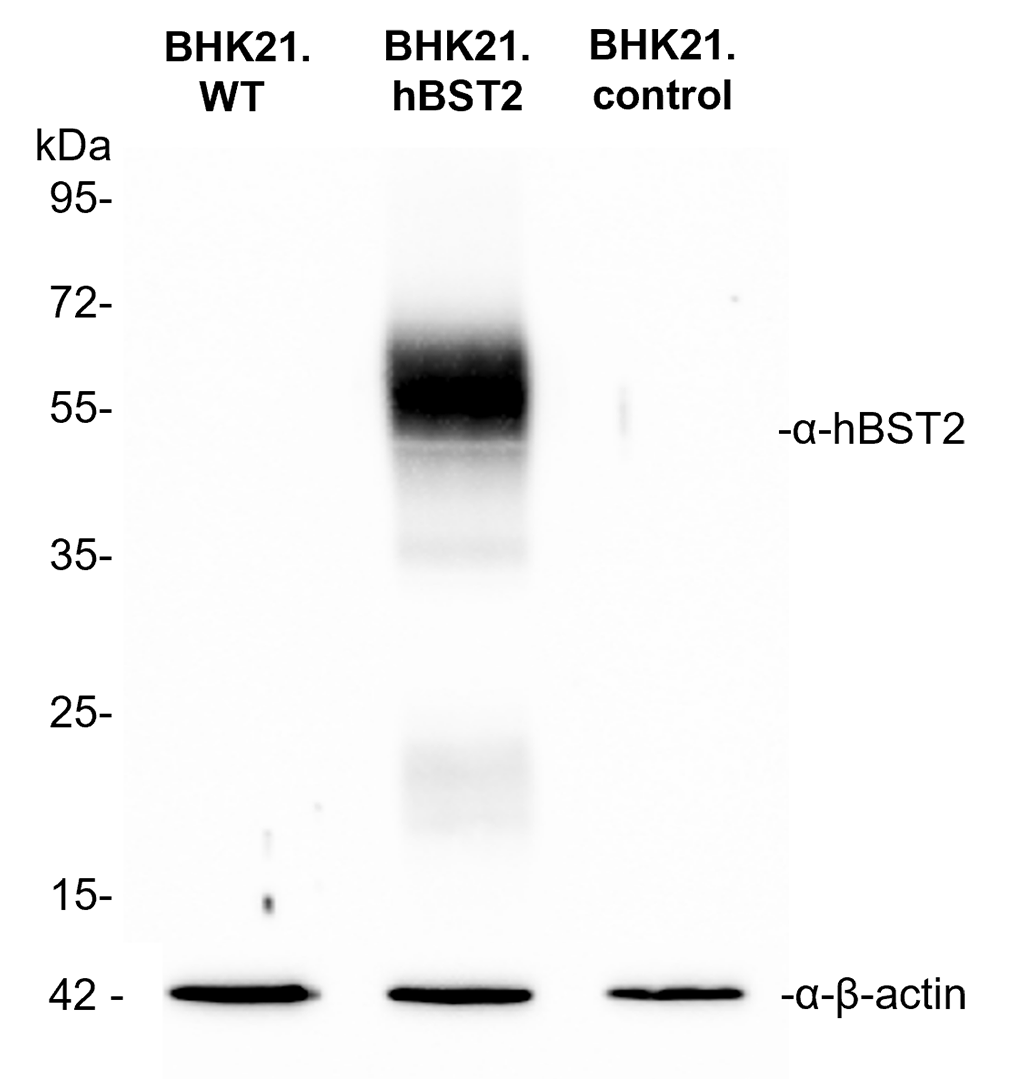

Supplement: S1 Fig — BHK21 cells including wild-type BHK21.C13 (BHK21.WT), BHK21 stably expressing hBST2 (BHK21.hBST2) and BHK21 control (transduced with control lentivirus) were harvested and subjected to western blot analysis. The membrane was probed with rabbit α-human BST2 antibody to detect hBST2 expression. (TIF) [file pone.0292833.s001.tif]

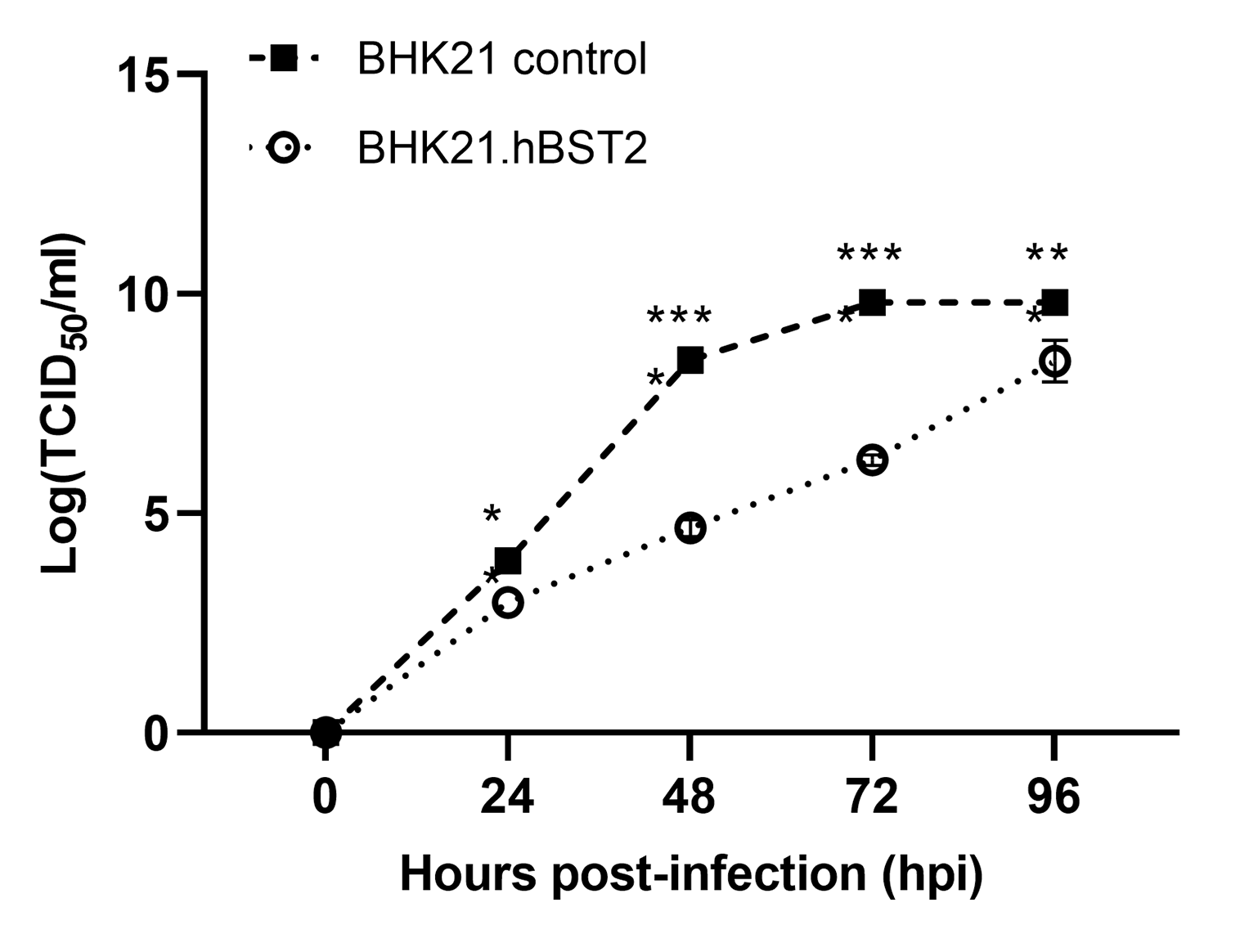

Supplement: S2 Fig — BHK21 stably expressing hBST2 (BHK21.hBST2) or BHK21 control (transduced with a control lentivirus) were infected with RABV CVS-11 strain at an MOI of 0.05. The supernatants were harvested for TCID50 assay. Error bars represent means ± SD of triplicates. Statistical significances were calculated by one-way ANOVA. **, p<0.01; ***, p<0.001; ****, p<0.0001. (TIF) [file pone.0292833.s002.tif]

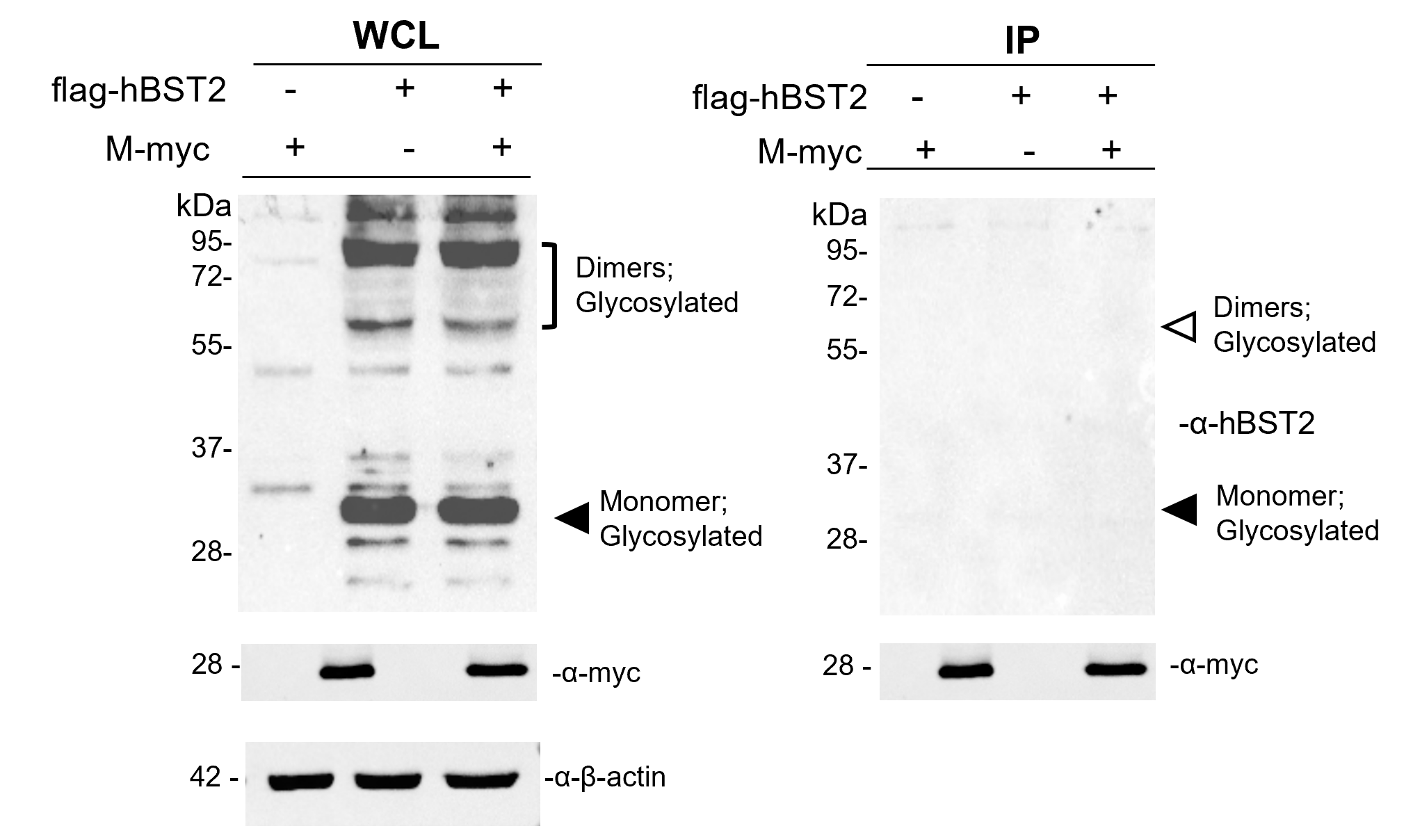

Supplement: S3 Fig — HEK293T cells were transfected with plasmids expressing flag-hBST2 and RABV M-myc. At 48 hpt, cell lysates were collected and immunoprecipitatated using mouse α-myc beads. Eluted proteins were probed with rabbit α-myc and α-flag antibodies to examine levels of RABV M and hBST2 using rabbit α-myc and α-flag antibodies, respectively. (TIF) [file pone.0292833.s003.tif]

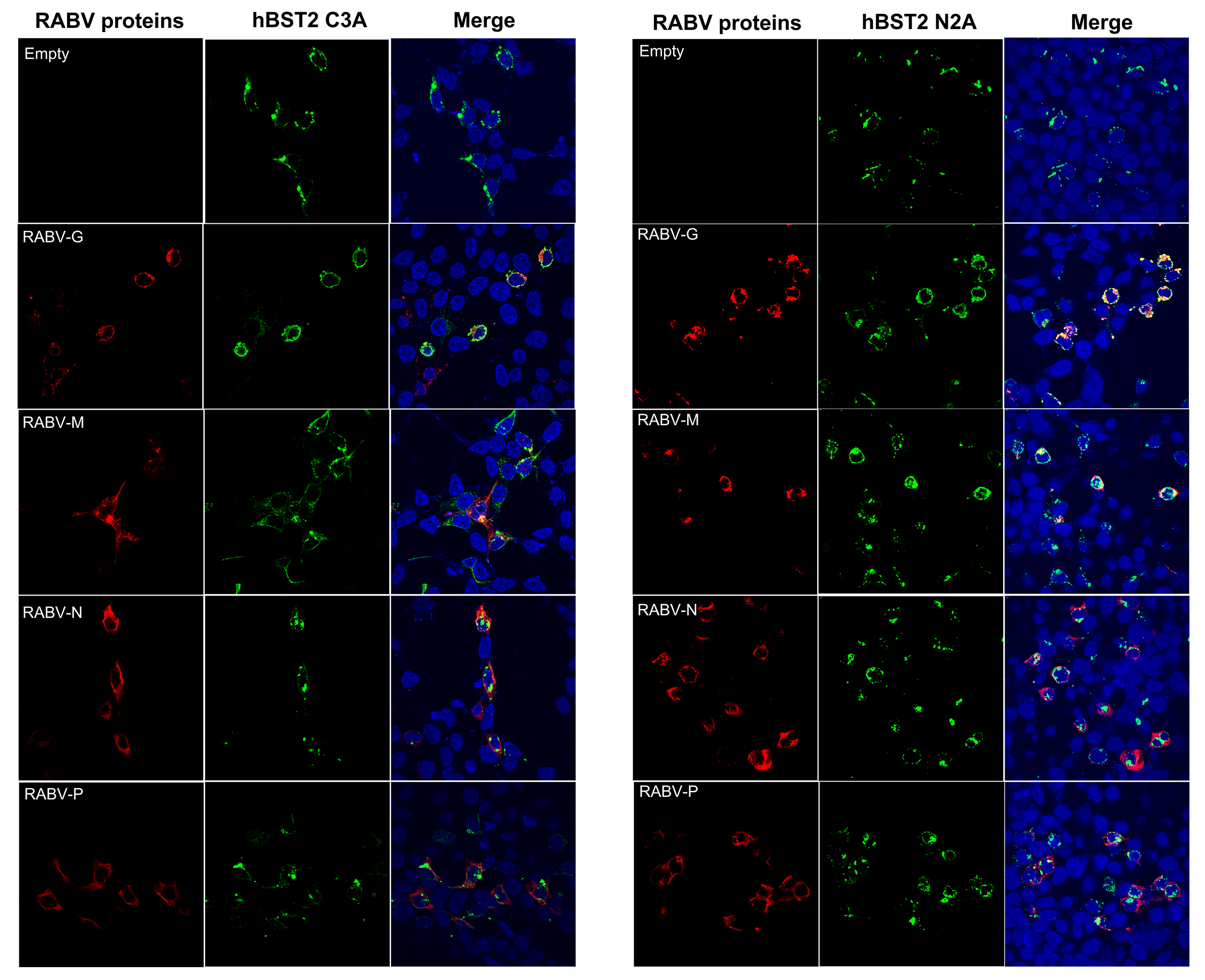

Supplement: S4 Fig — HEK293T cells were transfected with plasmids expressing individual myc-tagged RABV proteins and flag-tagged hBST2 C3A or N2A. At 24 hpt, cells were fixed and probed with mouse α-myc and rabbit α-flag primary antibodies to detect RABV proteins and hBST2 C3A or N2A, respectively, followed by Alexa Fluor® 488-conjugated goat α-rabbit IgG H&L and Alexa Fluor® 647-conjugated goat α-mouse IgG H&L secondary antibodies. Localization of the proteins was visualized by confocal microscopy. (TIF) [file pone.0292833.s004.tif]

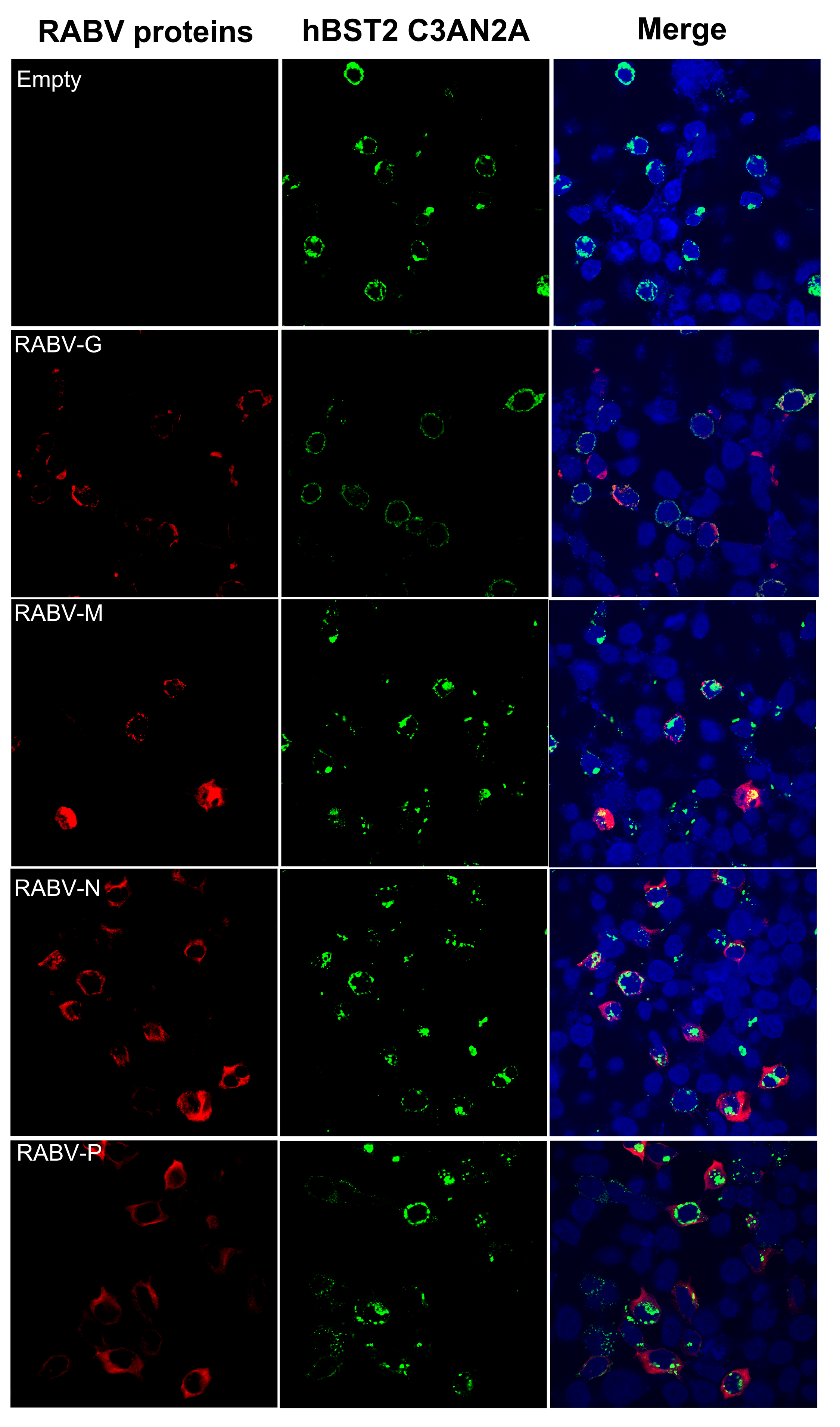

Supplement: S5 Fig — HEK293T cells were transfected with plasmids expressing individual myc-tagged RABV proteins and flag-tagged hBST2 C3AN2A. At 24 hpt, cells were fixed and probed with mouse α-myc and rabbit α-flag primary antibodies to detect RABV proteins and hBST2 C3AN2A, respectively, followed by Alexa Fluor® 488-conjugated goat α-rabbit IgG H&L and Alexa Fluor® 647-conjugated goat α-mouse IgG H&L secondary antibodies. Localization of the proteins was visualized by confocal microscopy. (TIF) [file pone.0292833.s005.tif]

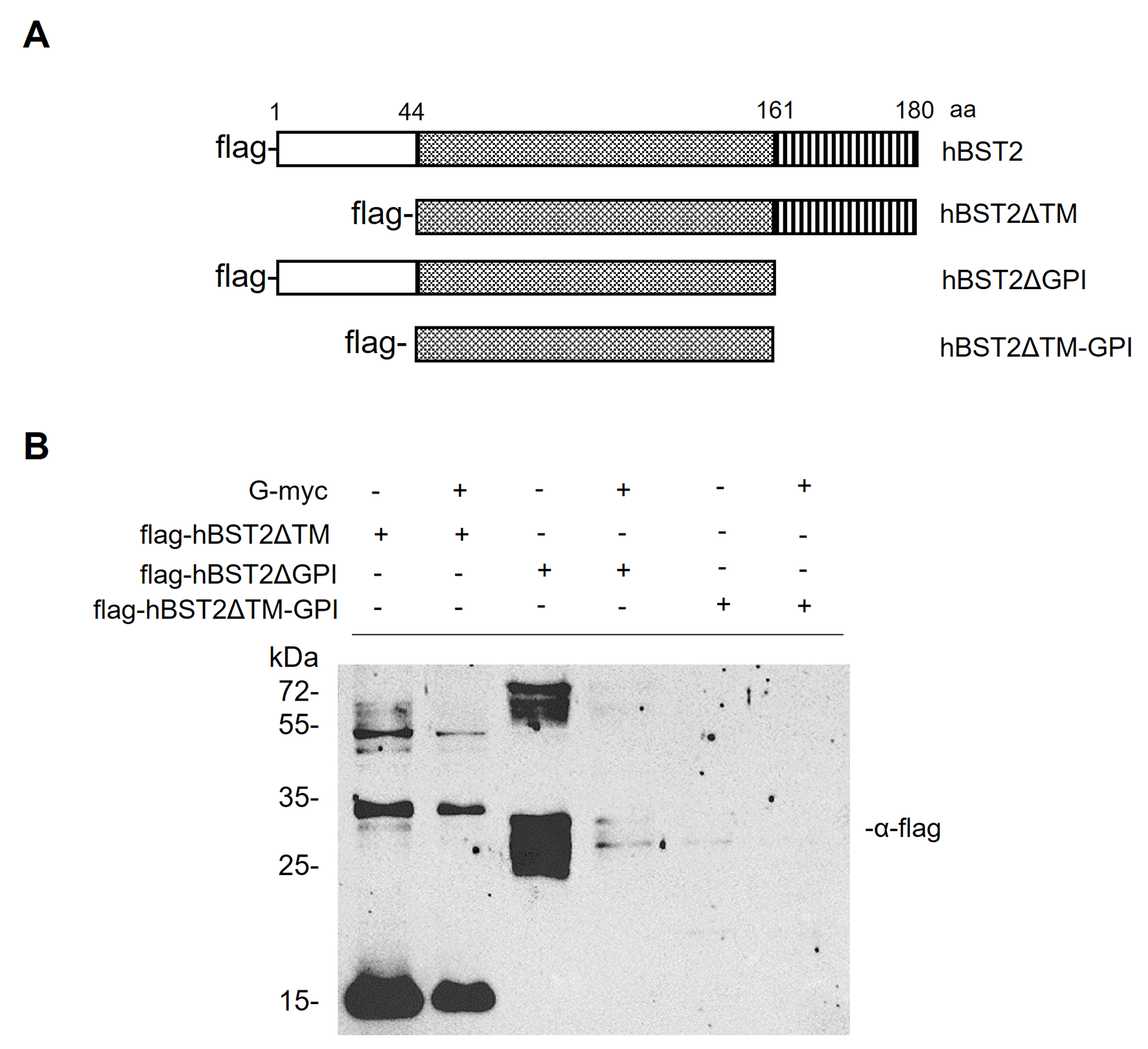

Supplement: S6 Fig — (A) A schematic representing hBST2 truncations include deletion of the N-terminal transmembrane domain (amino acids 1–43: ΔTM), deletion of the GPI anchor (amino acids 161–180: ΔGPI) and both (ΔTM-GPI). (B) HEK293T cells were transfected with the plasmids expressing each truncated hBST2 alone or with RABV-G. At 24 hpt, cell supernatants and pellets were harvested to examine protein expression. The cleared lysates were subjected to western blot analysis to examine hBST2 and RABV G using rabbit α-flag and rabbit α-myc antibodies, respectively. (TIF) [file pone.0292833.s006.tif]

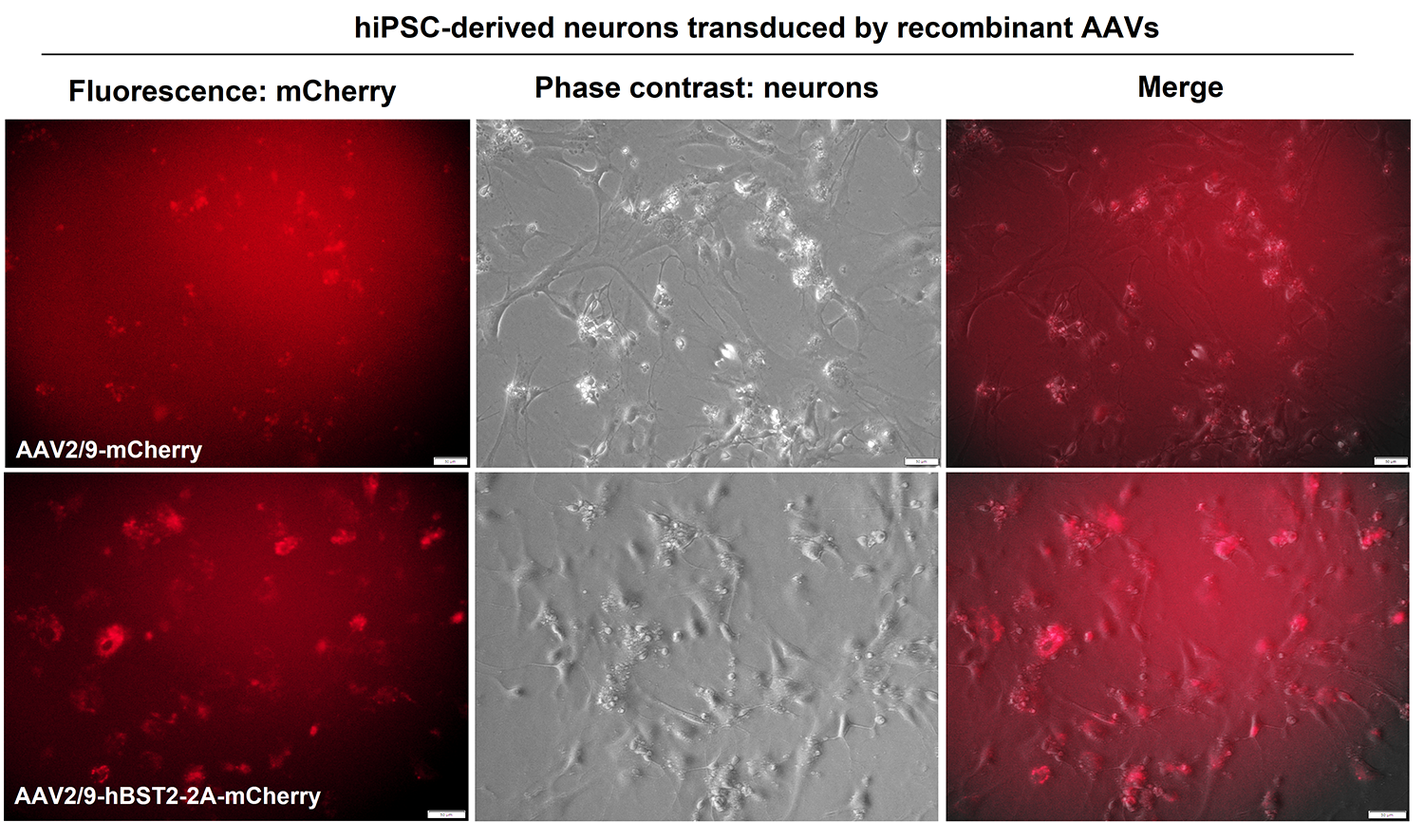

Supplement: S7 Fig — To generate recombinant AAV expressing hBST2, transfer vectors pAAV-hSyn1-hBST2-T2A-mcherry (T2A, self-cleaving peptides derived from Thosea asigna virus 2A, was used to cleave the fused hBST2-mcherry protein into two separated peptides) or pAAV-hSyn1-mcherry were constructed and co-transfected with a packaging plasmid (pAAV2/9n addgene: Plasmid #112865) and helper plasmid (pAdDeltaF6, addgene: Plasmid #112867) into HEK293A cells using PEI transfection. Transfected cells were maintained in OptiMEMTM media without FBS for 96 hours. Recombinant AAV in the supernatant was then rescued and purified through an Amicon® Ultra-15 Centrifugal Filter Unit. Concentrated virus was tittered by qPCR (genome copies/μl). hIPSC-derived neurons were transduced with each recombinant AAV at 104 genome copies for 5 days. The representative pictures present transduced neurons at 48 hours post transduction. (TIF) [file pone.0292833.s007.tif]
